# Synthesis of a New Class of Spirooxindole–Benzo[*b*]Thiophene-Based Molecules as Acetylcholinesterase Inhibitors

**DOI:** 10.3390/molecules25204671

**Published:** 2020-10-13

**Authors:** Assem Barakat, Saeed Alshahrani, Abdullah Mohammed Al-Majid, M. Ali, Mezna Saleh Altowyan, Mohammad Shahidul Islam, Abdullah Saleh Alamary, Sajda Ashraf, Zaheer Ul-Haq

**Affiliations:** 1 Department of Chemistry, College of Science, King Saud University, P.O. Box 2455, Riyadh 11451, Saudi Arabia; chemistry99y@gmail.com (S.A.); amajid@ksu.edu.sa (A.M.A.-M.); maly.c@ksu.edu.sa (M.A.); mislam@ksu.edu.sa (M.S.I.); alamary1401@yahoo.com (A.S.A.); 2 Department of Chemistry, Faculty of Science, Alexandria University, P.O. Box 426, Ibrahimia, Alexandria 21321, Egypt; 3 Department of Chemistry, College of Science, Princess Nourah Bint Abdulrahman University, Riyadh 11564, Saudi Arabia; msaltowyan@pnu.edu.sa; 4 Dr. Panjwani Center for Molecular medicine and Drug Research, International Center for Chemical and Biological Sciences, University of Karachi, Karachi 75270, Pakistan; sajda.ashraf@yahoo.com (S.A.); zaheer_qasmi@hotmail.com (Z.U.-H.)

**Keywords:** spirooxindole, benzo[*b*]thiophene, acetylcholinesterase inhibitory activity, molecular docking

## Abstract

A series of new oxindole-based spiro-heterocycles bearing the benzo[*b*]thiophene motif were synthesized via a 1,3-dipolar cycloaddition reaction and their acetylcholinesterase (AChE) inhibitory activity was evaluated. All the synthesized compounds exhibited moderate inhibitory activities against AChE, while **IIc** was found to be the most active analog with an IC_50_ value of 20,840 µM·L^−1^. Its molecular structure was a 5-chloro-substituted oxindole bearing benzo[*b*]thiophene and octahydroindole moieties. Based on molecular docking studies, **IIc** was strongly bound to the catalytic and peripheral anionic sites of the protein through hydrophilic, hydrophobic, and π-stacking interactions with Asp74, Trp86, Tyr124, Ser125, Glu202, Ser203, Trp236, Trp286, Phe297, Tyr337, and Tyr341. These interactions also indicated that the multiplicity of the **IIc** aromatic core significantly favored its activity.

## 1. Introduction

Alzheimer’s disease (AD) is one of the most common progressive neurodegenerative disorders, first identified by Alois Alzheimer in 1907. The main AD symptoms are cognitive decline and memory fragility [1]. Relevant global statistics have indicated that AD affects about 3% of elderly people aged between 65–74 [2]. Although the origin of this memory impairment has not yet been fully elucidated, many risk factors are considered to play a crucial role in developing AD including inflammation, oxidative stress, amyloid-β (Aβ) deposits, τ protein aggregation, and low acetylcholine (ACh) levels [3,4,5]. To date, several research teams have focused on the design and development of new molecules to target AD, while some FDA approved drugs involving the inhibition of cholinesterase (ChE) using various agents, such as donepezil, and galantamine have also been reported. However, these compounds have low therapeutic efficacy due to their short half-lives, low bioavailability, and high toxicity [6]. Therefore, there is still an urgent need to develop new, more potent, and less toxic lead compounds as ChE inhibitors (ChEIs).

**Figure 1:**
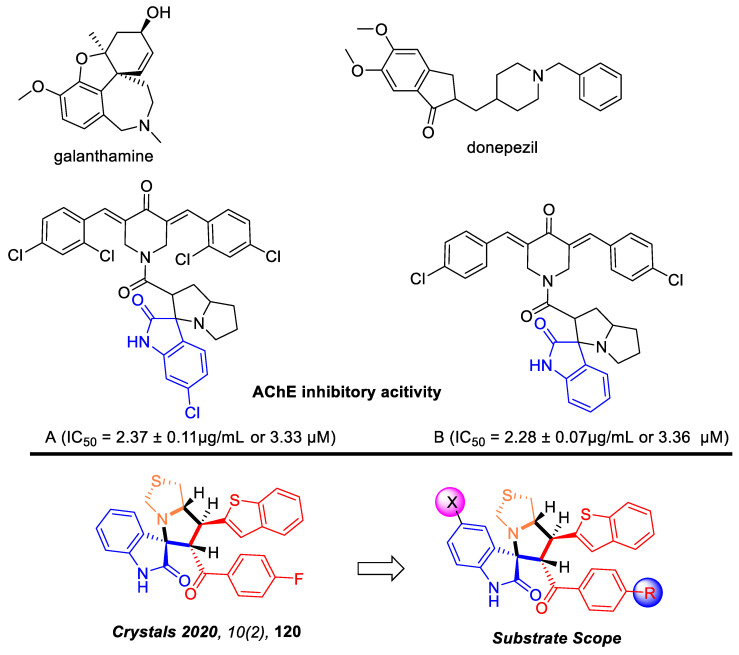
Representative oxindole-based spiro-heterocycles with significant acetylcholinesterase (AChE) inhibitory activity.

Oxindole-based spiro-heterocycles have been extensively studied due to their structure, which is suitable for various pharmacological targets, while several studies have reported their effective application as ChEIs [7,8,9,10,11,12,13,14]. Their structure has also been used to develop new lead compounds with improved solubility for drug discovery due to their expected binding efficiency in the target binding pockets [15,16,17]. Kia et al. have reported a series of oxindole-based spiro-heterocycles bearing pyrrolizine and piperidine moieties, which exhibited significant ACh esterase (AChE) inhibitory activity such as compound A (IC_50_ = 2.37 ± 0.11 µg/mL or 3.33 μM) (Figure 1) [7]. Further studies demonstrated that mono- and bis-spiro-pyrrolidines, such as compound B with an IC_50_ value of 1.68 ± 0.09 μM (Figure 1), have high potency against AChE [9].

Benzothiophene privilege structure is among the sulfur containing fused herterocycles which are interesting in drug discovery [18]. Many lead compounds having this nucleus possess divergent pharmaceutical activities, allowing them to act as anti-inflammatory, anti-cancer, anti-diabetic, anti-oxidant, anti-microbial, anti-convulsant agents, anti-tubercular, and many more [19,20,21,22,23,24,25,26].

Barakat et al. have also recently reported a compound prepared from an oxindole-based spiro-heterocycle and a benzo[*b*]thiophene moiety, which showed moderate activity against AChE (Figure 1) [12]. Here, we performed a structure–activity relationship study to further explore the extension and substrate scope of a new series of spirooxindole–benzo[*b*]thiophene-based analogs. Their AChE inhibitory activity was also assessed in vitro, while molecular modelling studies were performed to elucidate the structural features and interactions that favor the inhibitory activity of the examined compounds.

## 2. Results and Discussion

### 2.1. Synthesis of Analogs ***IIa***–***n***

**Scheme 1:**
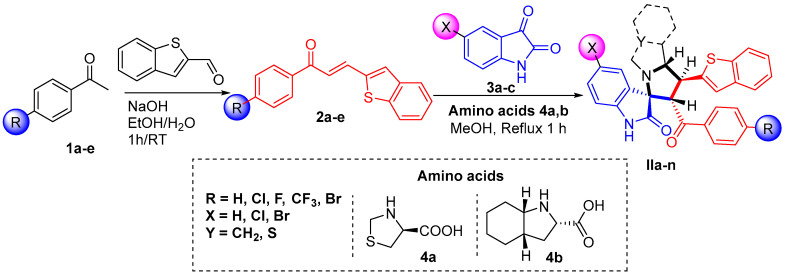
Synthesis of compounds **IIa**–**n**.

**Figure 2:**
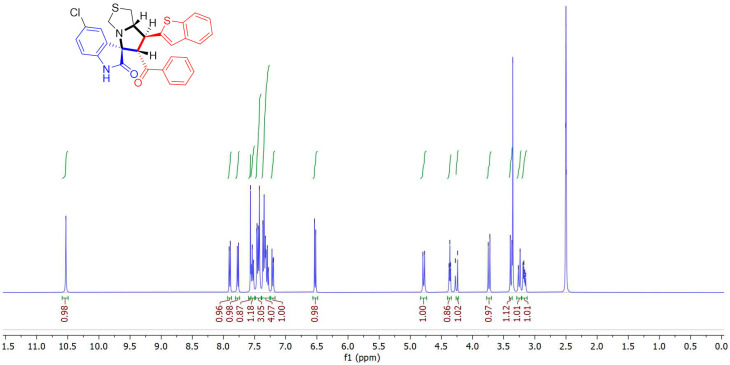

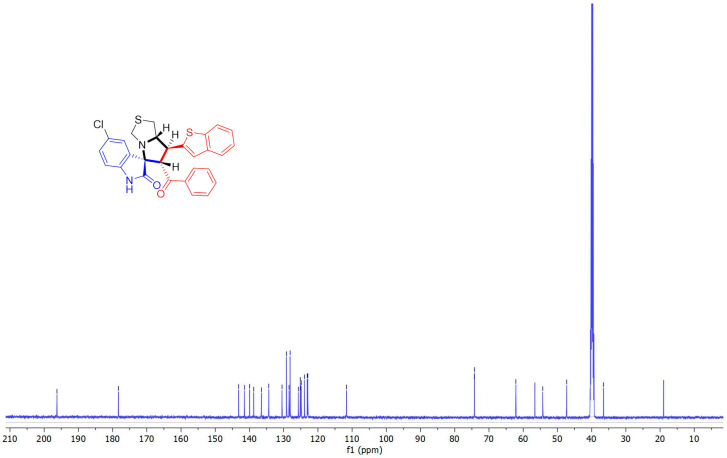
^1^H-NMR and ^13^C-NMR of **IIa** in DMSO-*d*_6_.

The general substrate scope of the synthetic compounds **IIa**–**n** is shown in Scheme 1. In particular, a series of spiro-oxindole-tethered benzo[*b*]thiophene scaffolds consisting of a single regio- and diastereo-selective isomer were synthesized from benzo[*b*]thiophene-based chalcones (**2a**–**e**), which were in turn prepared through an aldol condensation of the corresponding substituted acetophenones (**1a**–**e**) with benzo[*b*]thiophene-2-carboxaldehyde. Following the 1,3-dipolar cycloaddition reaction protocol [27,28,29,30,31,32,33,34], **2a**–**e** reacted with 5-substituted isatins (**3a**–**c**) and secondary amino acids, such as thioproline (**4a**) and octahydro-1*H*-indole-2-carboxylic acid (**4b**), forming the corresponding spiro-oxindole analogs **IIa–n** in high yields after purification by short column chromatography. The chemical features of the target compounds were assigned based on the NMR spectrum. As an example, the ^1^H-NMR spectrum of the **IIa** shows the characteristic peaks; the NH proton at δ 10.53 ppm; the aromatic protons in the region of δ 7.90–6.53 ppm; the protons of the fused bicyclic rings are shown in the chemical shift between δ 4.78–3.17 ppm as follows: the proton adjacent to benzoyl group 4.78 (d, *J* = 10.5 Hz, 1H, CHCO), C-H proton close to the benzothiophene ring at δ 4.37 (t, *J* = 5.1 Hz, 1H, CH), the four protons of the two CH_2_ groups appeared differently δ 4.24 (d, *J* = 10.8 Hz, 1H, CH_2_), δ 3.74 (d, *J* = 10.8 Hz, 1H, CH_2_), δ 3.25 (d, *J* = 11.4 Hz, 1H, CH_2_), δ 3.17 (*dd*, *J* = 11.6, 5.7 Hz, 1H, CH_2_); and the proton of the CHN shown at δ 3.38 (d, *J* = 10.4 Hz, 1H, CH). The ^13^C-NMR spectrum of the proposed carbon is perfectly shown in Figure 2.

### 2.2. In Vitro Biological Activity

The in vitro cholinesterase inhibitory activity of the 14 synthesized spiro-oxindole derivatives (**IIa**–**n**) were evaluated [9]. As shown in Table 1, all analogs exhibited AChE activity with IC_50_ values ranging between 20,840–121,690 µM·L^−1^. Among them, analogs **IIc**, **IId**, **IIf**, **IIg**, **IIl** and **IIn** showed the highest inhibitory activity with an IC_50_ value of 20,840; 37,670; 34,020; 23,040; 29,760 and 36,830 µM L^−1^ respectively, while **IIc** with a 5-chloro-substituted oxindole structure bearing benzo[*b*]thiophene and octahydroindole moieties, was the most active in this group. The second group of spiro-oxindole analogs (**IIe**, **IIk**, and **IIm**) with an IC_50_ value of 50,590; 41,530 and 41,450 μM L^−1^ respectively showed moderate activity, comparable to that of the positive control. In contrast, the last group included analogs **IIa**, **IIb**, and **IIh**–**IIj**, which showed weak AChE inhibitory activity with an IC_50_ value of more than 70,000 μM L^−1^. Galantamine was used as a positive control for comparison.

### 2.3. Molecular Docking Study

**Figure 3:**
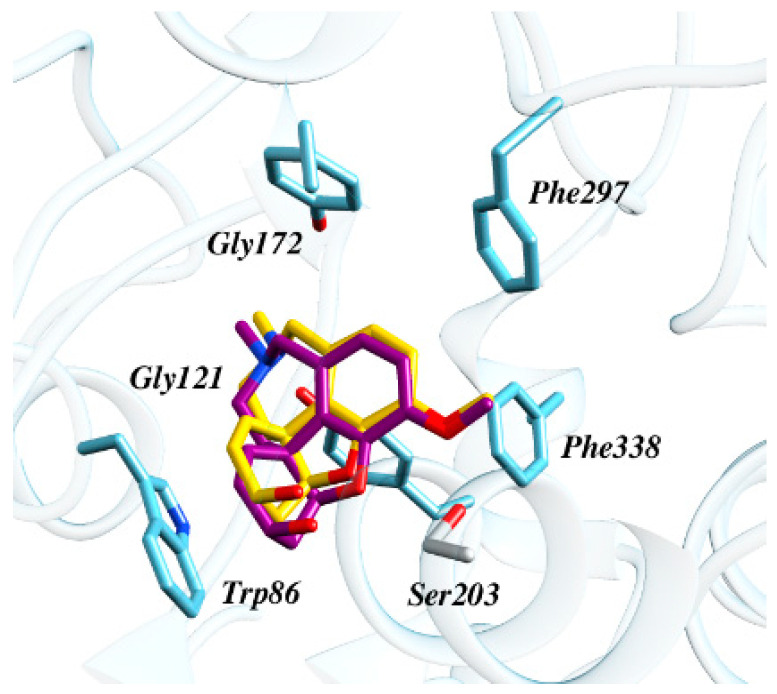
Re-docking of a galantamine molecule. Galantamine (PDB ID: 4EY6) is indicated with yellow color and its docked confirmation is presented in magenta. Ligand heavy atom root-mean-square deviation (RMSD) = 0.81 Å.

**Figure 4:**
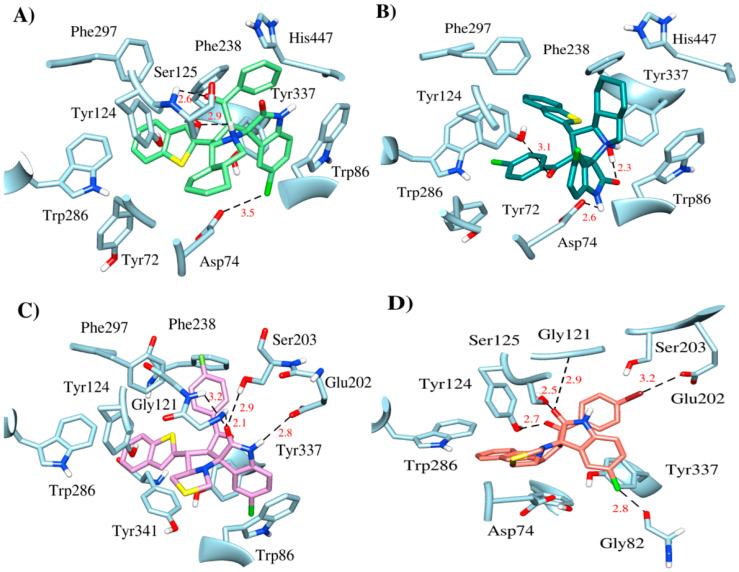
Visual presentation of the binding patterns of the spiro-benzothiophene derivatives (**A**) **IIc**, (**B**) **IIf**, (**C**) **IIg**, and (**D**) **IIl**. The dotted lines indicate the intramolecular hydrogen bonds.

In order to identify the binding pattern and mechanism of the synthesized spiro-benzothiophene derivatives, the complex AChE enzyme was studied using molecular docking based on the atomic coordinates of the crystallographic structure of galantamine. All analogs were docked using the default MOE docking protocol and the obtained conformations were visually analyzed to elucidate the existing interactions. The binding affinity of the standard reference galantamine, was attributed to the presence of hydroxyl groups that might help its stabilization in the binding pocket through classical and non-classical hydrogen bonds with Ser203 and Tyr337 (Figure 3). The docking results indicated that the conformations with the highest score (Table 2) of all the derivatives fitted well into the binding cavity of the AChE enzyme by developing different interactions with the active site residues Asp74, Gly82, Thr83, Trp86, Gly121, Glu202, Ser203, Tyr337, Tyr341, and His447. However, compounds bearing electronegative substituents on the benzene ring developed weak hydrophobic interactions with the surrounding residues. Moreover, bulky substituents at the R-position could reduce the compounds’ activity due to steric hindrance. Thus, the elucidation of the structural features and the effect of different substituents on the spiro-benzothiophene derivatives, such as the presence of electron-donating (H and NH_2_) or electron-withdrawing (halogen and haloalkyl) groups, explained the effective inhibition of the AChE enzyme. As depicted in Figure 4A, the most active compound (**IIc**) with an IC_50_ value of 20,840 µML^−1^ was strongly bound to the catalytic and peripheral anionic sites of the protein through hydrophobic and π-stacking interactions with Tyr72, Asp74, Trp86, Tyr124, Trp286, Phe297, Tyr337, Phe338, and Tyr341. These interactions further indicated the beneficial effect of the aromatic core multiplicity on the compound’s high activity. Hydrophilic interactions were also observed between the hydrogen-bond donor of Ser125 and the carbonyl group of **IIc** at a distance of 2.6 Å. Their interaction was further stabilized by a special halogen bond interaction between the 5-Cl atom and Asp74.

Compounds **IIf**, **IIg**, and **IIl** also exhibited moderate activities in the range of 23,040–34,020 µM L^−1^. Except for a few interactions, their molecular docking images were similar to the binding mode of **IIc**. Compound **IIf**, bearing a Cl deactivating group on the phenyl ring, showed less binding affinity than **IIc** bearing a hydrogen atom on the same phenyl ring position. Moreover, the side chain of the Asp74, Tyr124, and Tyr337 residues developed hydrophilic interactions with the carbonyl and NH_2_ groups of the **IIf** indoline ring at distances of 2.6, 3.1, and 2.3 Å, respectively (Figure 4B). Furthermore, as shown in Figure 4C,D, the proposed binding mode of compounds **IIg** and **IIl** was very similar. In particular, most interactions were located in the region of the Trp86, Tyr124, Trp286, Phe295, Phe297, Tyr337, Phe338, Tyr341, and Tyr449 amino acid residues due to development of hydrophobic and π interactions at the catalytic anionic site and the formation of hydrogen bonds with Tyr124, Ser125, Gly121, and Ser203 at the edge of the peripheral region. However, the carbonyl and NH_2_ groups of the **IIg** indoline ring developed hydrophilic interactions with Gly121, Ser125, Glu202, and Ser203 at distances of 2.1, 3.2, 2.9, and 2.8 Å, respectively, whereas the hydrophilic interactions of **IIl** with Gly121, Ser125, and Glu202 were observed at distances of 2.7, 2.5, and 2.9 Å, respectively. Moreover, the halogen bond formed with Gly82 and Ser203 further enhanced the binding of **IIl.** Therefore, the docking results of the synthesized spiro-benzothiophene derivatives were in good agreement with the experimental findings, providing significant information about their binding mechanism to AChE.

## 3. Materials and Methods

### 3.1. General Experimental Information

All the chemicals were purchased from Sigma–Aldrich (Riedstraße, Germany), and Fluka (Buchs, Switzerland), and were used without further purification, unless otherwise reported. The melting points were measured on a Gallenkamp melting point apparatus (Bibby Scientific Limited, Beacon Road, Stone, Staffordshire, UK) in open glass capillaries and are not corrected. The infrared (IR) spectra were measured as KBr pellets on a Nicolet 6700 Fourier-transform IR spectrophotometer (Thermo Fisher Scientific, Madison, WI, USA). The ^1^H (400 MHz) and ^13^C (100 MHz) nuclear magnetic resonance (NMR) spectra were recorded on a Varian Mercury Jeol-400 NMR spectrometer (Tokyo, Japan) in CDCl_3_ or DMSO-*d*_6_. The chemical shifts (δ) are provided in ppm and the *J* coupling constants in Hz. The mass spectra were recorded on a JEOL JMS-600 H mass spectrometer (Santa Clara, CA, USA). while the elemental analysis of the synthesized compounds was performed using an Elmer 2400 Elemental Analyzer (CHN mode) (Perkin Elmer, Waltham, MA, USA). The AChE assay and molecular docking protocols are described in the supporting information.

### 3.2. General Procedure for the Synthesis of Chalcones ***2a***–***e***

The chalcone derivatives **2a**–**e** were synthesized based on a reported procedure [27,28] using benzo[*b*]thiophene-2-carboxaldehyde (1.0 eq.) in ethanol and the corresponding substituted acetophenone (acetopehnone, *p*-Cl- acetopehnone, *p*-Br-acetopehnone, *p*-F-acetopehnone and *p*-CF_3_-acetopehnone) (1.0 eq.) (**1a**–**e**) in the presence of aqueous NaOH.

*(E)-3-(Benzo[b]thiophen-2-yl)-1-phenylprop-2-en-1-one* (**2a**). The spectrum is consistent with the reported literature [36,37].

*(E)-3-(Benzo[b]thiophen-2-yl)-1-(4-chlorophenyl)prop-2-en-1-one* (**2b**). ^1^H-NMR (400 MHz, CDCl_3_) δ: 7.30 (d, 1H, *J* = 15.24 Hz, CH=CH), 7.44–7.36 (m, 2H, Ar–H), 7.49 (d, 1H, *J* = 8.16 Hz, Ar–H), 7.56 (s, 1H, C=CH), 7.81 (dd, 2H, *J* = 10.6, 7.84 Hz, Ar–H), 7.97 (d, 2H, *J* = 8.08 Hz, Ar–H), 8.05 (d, 1H, *J* = 15.28 Hz, CH=CH); ^13^C-NMR (100 MHz, CDCl_3_) δ: 188.4, 140.4, 140.2, 139.7, 139.5, 136.3, 130.1, 129.9, 129.3, 128.9, 124.9.

*(E)-3-(Benzo[b]thiophen-2-yl)-1-(4-fluorophenyl)prop-2-en-1-one* (**2c**). ^1^H-NMR (400 MHz, CDCl_3_) δ: 7.18 (t, 1H, *J* = 15.24 Hz, Ar–H), 7.35 (d, 1H, *J* = 15.24 Hz, CH=CH), 7.42–7.35 (m, 2H, Ar–H), 7.49 (d, 1H, *J* = 8.16 Hz, Ar–H), 7.57 (s, 1H, C=CH), 7.82 (dd, 2H, *J* = 10.6, 7.84 Hz, Ar–H), 8.06 (d, 2H, *J* = 8.08 Hz, Ar–H), 8.08 (d, 1H, *J* = 15.28 Hz, CH=CH); ^13^C-NMR (100 MHz, CDCl_3_) δ: 188.0, 140.3, 140.2, 139.7, 134.4, 134.3, 131.2, 122.6, 115.8.

*(E)-3-(Benzo[b]thiophen-2-yl)-1-(4-(trifluoromethyl)phenyl)prop-2-en-1-one* (**2d**). ^1^H-NMR (400 MHz, CDCl_3_) δ: 7.30 (d, 1H, *J* = 15.24 Hz, CH=CH), 7.43–7.37 (m, 2H, Ar–H), 7.49 (d, 1H, *J* = 8.16 Hz, Ar–H), 7.60 (s, 1H, C=CH), 7.83 (dd, 2H, J = 10.6, 7.84 Hz, Ar–H), 8.07 (d, 2H, *J* = 8.08 Hz, Ar–H), 8.12 (d, 1H, *J* = 15.28 Hz, CH=CH); ^13^C-NMR (100 MHz, CDCl_3_) δ: 188.8, 140.9, 140.5, 139.9, 139.7, 130.8, 128.8, 126.9, 125.8, 125.1, 124.8, 122.4.

*(E)-3-(Benzo[b]thiophen-2-yl)-1-(4-bromophenyl)prop-2-en-1-one* (**2e**). ^1^H-NMR (400 MHz, CDCl_3_) δ: 7.25 (d, 1H, *J* = 15.24 Hz, CH=CH), 7.41–7.37 (m, 2H, Ar–H), 7.49 (d, 1H, *J* = 8.16 Hz, Ar–H), 7.58 (s, 1H, C=CH), 7.82 (dd, 2H, *J* = 10.6, 7.84 Hz, Ar–H), 7.90 (d, 2H, *J* = 8.08 Hz, Ar–H), 8.05 (d, 1H, *J* = 15.28 Hz, CH=CH); ^13^C-NMR (100 MHz, CDCl_3_) δ: 188.6, 140.4, 140.2, 139.7, 136.8, 130.1, 129.9, 129.3, 128.9, 124.9.

### 3.3. General Procedure for the Synthesis of Oxindole-Based Spiro-Heterocycles ***IIa***–***n***

The oxindole-based spiro-heterocycles **IIa**–**n** were synthesized through an one-pot reaction using equimolar amounts of each chalcone (**2a**–**e**), amino acid (**4a**–**b**) (1.0 mmol), and substituted isatin (**3a–c**, 1.0 mmol), which were refluxed in methanol (10 mL) for 1–3 h. All analogs were obtained as precipitates, which were filtered and washed with a small amount of MeOH. The final product were separated in faint yellow color.

*(3S)-7′-(Benzo[b]thiophen-2-yl)-6′-benzoyl-5-chloro-3′,6′,7′,7a’-tetrahydro-1′H-spiro[indoline-3,5′-pyrrolo[1,2-c]thiazol]-2-one* (**IIa**). Analog **IIa** was synthesized using **2a** (264 mg), 5-chloro-isatin (**3b**) (181 mg), and thioproline **4a** (133 mg). Yield: 470 mg (0.91 mmol, 91%); m.p: 121 °C; ^1^H-NMR (400 MHz, DMSO-*d*_6_) δ: 10.53 (s, 1H, NH), 7.90 (d, *J* = 7.7 Hz, 1H, Ar–H), 7.77 (d, *J* = 8.0 Hz, 1H, Ar–H), 7.57 (s, 1H, Ar–H), 7.54 (t, *J* = 7.4 Hz, 1H, Ar–H), 7.49–7.39 (m, 3H, Ar–H), 7.32 (dt, *J* = 19.8, 7.6 Hz, 4H, Ar–H), 7.21 (dd, *J* = 8.5, 1.7 Hz, 1H, Ar–H), 6.53 (d, *J* = 8.1 Hz, 1H, Ar–H), 4.78 (d, *J* = 10.5 Hz, 1H, CHCO), 4.37 (t, *J* = 5.1 Hz, 1H, CH), 4.24 (d, *J* = 10.8 Hz, 1H, CH_2_), 3.74 (d, *J* = 10.8 Hz, 1H, CH_2_), 3.38 (d, *J* = 10.4 Hz, 1H, CH), 3.25 (d, *J* = 11.4 Hz, 1H, CH_2_), 3.17 (dd, *J* = 11.6, 5.7 Hz, 1H, CH_2_); ^13^C-NMR (126 MHz, DMSO-*d*_6_) δ: 196.26, 178.30, 143.15, 141.45, 139.96, 138.74, 136.52, 134.37, 130.47, 129.17, 128.42, 128.11, 125.69, 125.14, 125.06, 124.86, 123.91, 123.10, 122.94, 111.65, 74.27, 74.24, 62.14, 54.28, 47.31, 36.51; IR (KBr, cm^−1^) *ν*_max_ = 1475, 1548, 1605, 1705, 2915, 3100, 3265; [Anal. Calcd. for C_28_H_21_ClN_2_O_2_S_2_: C, 65.04; H, 4.09; N, 5.42; Found: C, 64.93; H, 4.21; N, 5.65]; LC/MS (ESI, *m/z*): 517.10 [M^+^].

*(3S)-7′-(Benzo[b]thiophen-2-yl)-6′-benzoyl-3′,6′,7′,7a’-tetrahydro-1′H-spiro[indoline-3,5′-pyrrolo[1,2-c]thiazol]-2-one* (**IIb**). Analog **IIb** was prepared using **2a** (264 mg), isatin (**3a**) (147 mg), and thioproline (**4a**) (133 mg). Yield: 443 mg (0.92 mmol, 92%); m.p: 65 °C; ^1^H-NMR (400 MHz, DMSO-*d*_6_) δ: 10.39 (s, 1H, NH), 7.90 (d, *J* = 7.8 Hz, 1H, Ar–H), 7.77 (d, *J* = 7.8 Hz, 1H, Ar–H), 7.52 (d, *J* = 12.4 Hz, 2H, Ar–H), 7.44–7.24 (m, 7H, Ar–H), 7.13 (s, 1H, Ar–H), 6.95 (s, 1H, Ar–H), 6.51 (d, *J* = 7.8 Hz, 1H, Ar–H), 4.76 (s, 1H, CHCO), 4.27 (d, *J* = 8.7 Hz, 2H, CH), 3.73 (d, *J* = 10.2 Hz, 1H, CH_2_), 3.34 (d, *J* = 10.2 Hz, 1H, CH), 3.18 (t, *J* = 2.8 Hz, 2H, CH_2_); ^13^C-NMR (101 MHz, DMSO-*d*_6_) δ: 196.36, 178.65, 143.65, 142.63, 140.07, 138.82, 136.80, 134.07, 130.53, 129.04, 128.53, 128.11, 125.13, 124.82, 123.91, 123.23, 122.99, 121.67, 110.20, 74.3, 73.90, 62.45, 54.00, 47.20, 36.51; IR (KBr, cm^−1^) *ν*_max_ = 1485, 1548, 1610, 1718, 2930, 3135, 3285; [Anal. Calcd. for C_28_H_22_N_2_O_2_S_2_: C, 69.68; H, 4.59; N, 5.80; Found: C, 69.79; H, 4.47; N, 6.01]; LC/MS (ESI, *m/z*): 483.20 [M^+^].

*(3S)-1′-(Benzo[b]thiophen-2-yl)-2′-benzoyl-5-chloro-1′,2′,5′,5a’,6′,7′,8′,9′,9a’,9b’-decahydrospiro[indoline-3,3′-pyrrolo[2,1-a]isoindol]-2-one* (**IIc**). Analog **IIc** was prepared using **2a** (264 mg), 5-chloro-isatin (**3b**) (181 mg), and (2*S*,3a*S*,7a*S*)-octahydro-1*H*-indole-2-carboxylic acid **4b** (169 mg). Yield: 458 mg (0.83 mmol, 83%); m.p: 110 °C; ^1^H-NMR (400 MHz, DMSO-*d*_6_) δ: 10.27 (s, 1H), 7.87 (d, *J* = 7.9 Hz, 1H, Ar–H), 7.74 (d, *J* = 7.8 Hz, 1H, Ar–H), 7.52 (d, *J* = 7.1 Hz, 1H, Ar–H), 7.48–7.24 (m, 8H, Ar–H), 7.18 (d, *J* = 8.2 Hz, 1H, Ar–H), 6.48 (d, *J* = 8.2 Hz, 1H, Ar–H), 4.93 (d, *J* = 11.7 Hz, 1H, CHCO), 4.34 (t, *J* = 10.8 Hz, 1H, CH), 4.27–4.18 (m, 1H, CH), 3.17 (d, *J* = 3.7 Hz, 1H, CH), 2.21–2.01 (m, 2H, CH_2_), 1.71 (dd, *J* = 11.2, 6.0 Hz, 1H, CH_2_), 1.51 (ddd, *J* = 14.1, 9.6, 4.4 Hz, 2H, CH_2_), 1.33 (*p*, *J* = 11.8, 10.7 Hz, 2H, CH_2_), 1.09 (dd, *J* = 10.9, 6.1 Hz, 1H, CH_2_), 1.04–0.93 (m, 1H, CH_2_), 0.88 (tt, *J* = 13.3, 3.8 Hz, 1H, CH_2_), 0.77–0.65 (m, 1H, CH_2_); ^13^C-NMR (101 MHz, DMSO-*d*_6_) δ: 196.53, 180.04, 144.01, 141.23, 140.09, 138.74, 136.97, 134.08, 129.87, 129.07, 128.33, 128.19, 125.94, 125.78, 124.96, 124.52, 123.72, 122.86, 121.87, 111.38, 71.90, 70.94, 65.17, 57.38, 48.71, 41.92, 36.70, 28.05, 27.96, 25.00, 19.70; IR (KBr, cm^−1^) *ν*_max_ = 1480, 1555, 1608, 1725, 2920, 31,125, 3285; [Anal. Calcd. for C_33_H_29_ClN_2_O_2_S: C, 71.66; H, 5.28; N, 5.06; Found: C, 71.49; H, 5.13; N, 5.22]; LC/MS (ESI, *m/z*): 553.20 [M^+^].

*(3S)-7′-(Benzo[b]thiophen-2-yl)-5-chloro-6′-(4-chlorobenzoyl)-3′,6′,7′,7a’-tetrahydro-1′H-spiro[indoline-3,5′-pyrrolo[1,2-c]thiazol]-2-one* (**IId**). Analog **IId** was obtained using **2b** (298 mg), 5-chloro-isatin (**3b**) (181 mg), and thioproline (**4a**) (133 mg). Yield: 478 mg (0.87 mmol, 87%); m.p: 60 °C; ^1^H-NMR (400 MHz, DMSO-*d*_6_) δ: 10.55 (s, 1H, NH), 7.91 (d, *J* = 7.9 Hz, 1H, Ar–H), 7.77 (d, *J* = 7.4 Hz, 1H, Ar–H), 7.58 (s, 1H, Ar–H), 7.44 (dt, *J* = 14.4, 6.2 Hz, 5H, Ar–H), 7.33 (dt, *J* = 18.4, 7.3 Hz, 2H, Ar–H), 7.26–7.19 (m, 1H, Ar–H), 6.56 (d, *J* = 8.2 Hz, 1H, Ar–H), 4.76 (d, *J* = 10.9 Hz, 1H, CHCO), 4.30–4.17 (m, 2H, CH_2_), 3.74 (d, *J* = 10.4 Hz, 1H, CH), 3.39 (d, *J* = 10.6 Hz, 1H, CH), 3.26 (d, *J* = 11.4 Hz, 1H, CH_2_), 3.17 (dd, *J* = 11.7, 5.9 Hz, 1H, CH_2_); ^13^C-NMR (126 MHz, DMSO-*d*_6_) δ: 195.47, 178.04, 143.07, 141.49, 139.99, 139.26, 138.79, 135.26, 130.57, 130.00, 129.31, 128.39, 125.71, 125.12, 124.99, 124.83, 123.89, 123.13, 122.96, 111.66, 74.33, 74.20, 62.43, 54.28, 47.02, 36.52; IR (KBr, cm^−1^) *ν*_max_ = 1485, 1498, 1534, 1634, 1726, 2934, 3088, 3288; [Anal. Calcd. for C_28_H_20_Cl_2_N_2_O_2_S_2_: C, 60.98; H, 3.66; N, 5.08; Found: C, 61.14; H, 3.52; N, 5.24]; LC/MS (ESI, *m/z*): 551.10 [M^+^].

*(3S)-7′-(Benzo[b]thiophen-2-yl)-6′-(4-chlorobenzoyl)-3′,6′,7′,7a’-tetrahydro-1′H-spiro[indoline-3,5′-pyrrolo[1,2-c]thiazol]-2-one* (**IIe**). Analog **IIe** was prepared using **2b** (298 mg), isatin (**3a**) (147 mg), and thioproline (**4a**) (133 mg). Yield 480 mg (0.93 mmol, 93%); m.p: 128 °C; ^1^H-NMR (400 MHz, DMSO-*d*_6_) δ: 10.40 (s, 1H, NH), 7.90 (d, *J* = 7.9 Hz, 1H, Ar–H), 7.77 (d, *J* = 7.9 Hz, 1H, Ar–H), 7.55 (s, 1H, Ar–H), 7.45–7.37 (m, 3H, Ar–H), 7.36–7.27 (m, 4H, Ar–H), 7.15 (t, *J* = 7.7 Hz, 1H, Ar–H), 6.96 (t, *J* = 7.5 Hz, 1H, Ar–H), 6.53 (d, *J* = 7.8 Hz, 1H, Ar–H), 4.74 (d, *J* = 10.8 Hz, 1H, CHCO), 4.32–4.19 (m, 2H, CH_2_), 3.73 (d, *J* = 10.3 Hz, 1H, CH), 3.36 (d, *J* = 10.3 Hz, 1H, CH), 3.19 (d, *J* = 5.7 Hz, 2H, CH_2_); ^13^C-NMR (126 MHz, DMSO-*d*_6_) δ: 195.56, 178.46, 143.41, 142.55, 140.02, 138.95, 138.78, 135.44, 130.62, 129.89, 129.14, 128.43, 125.10, 124.79, 123.87, 123.04, 122.94, 122.89, 121.69, 110.22, 74.33, 73.90, 62.60, 54.05, 46.95, 36.50; IR (KBr, cm^−1^) *ν*_max_ = 1485, 1545, 1615, 1715, 2920, 3115, 3275; [Anal. Calcd. for C_28_H_21_ClN_2_O_2_S_2_: C, 65.04; H, 4.09; N, 5.42; Found: C, 65.27; H, 4.16; N, 5.59]; LC/MS (ESI, *m/z*): 517.20 [M^+^].

*(3S)-1′-(Benzo[b]thiophen-2-yl)-5-chloro-2′-(4-chlorobenzoyl)-1′,2′,5′,5a’,6′,7′,8′,9′,9a’,9b’-decahydrospiro[indoline-3,3′-pyrrolo[2,1-a]isoindol]-2-one***(IIf**). Analog **IIf** was prepared using **2b** (298 mg), 5-chloro-isatin (**3b**) (181 mg), and (2*S*,3a*S*,7a*S*)-octahydro-1*H*-indole-2-carboxylic acid (**4b**) (169 mg). Yield: 504 mg (0.86 mmol, 86%); m.p: 119 °C; ^1^H-NMR (400 MHz, DMSO-*d*_6_) δ: 10.29 (s, 1H, NH), 7.87 (d, *J* = 8.0 Hz, 1H, Ar–H), 7.74 (d, *J* = 7.6 Hz, 1H, Ar–H), 7.49–7.34 (m, 6H Ar–H), 7.29 (dd, *J* = 11.2, 7.4 Hz, 2H Ar–H), 7.19 (d, *J* = 8.4 Hz, 1H Ar–H), 6.51 (d, *J* = 8.6 Hz, 1H Ar–H), 4.91 (d, *J* = 11.6 Hz, 1H, CHCO), 4.33 (t, *J* = 10.8 Hz, 1H, CH), 4.22 (t, *J* = 7.5 Hz, 1H, CH), 3.16 (d, *J* = 3.7 Hz, 1H, CH), 2.11 (*q*, *J* = 9.1, 8.2 Hz, 2H, CH_2_), 1.70 (dd, *J* = 11.1, 6.1 Hz, 1H, CH_2_), 1.50 (s, 2H, CH_2_), 1.40–1.22 (m, 2H, CH_2_), 1.05–0.93 (m, 1H, CH_2_), 0.90–0.78 (m, 2H, CH_2_), 0.70 (d, *J* = 13.8 Hz, 1H, CH_2_); ^13^C-NMR (101 MHz, DMSO-*d*_6_) δ: 195.72, 179.93, 143.84, 141.17, 140.09, 139.03, 138.77, 135.64, 130.03, 129.23, 128.53, 125.90, 125.82, 124.97, 124.54, 123.80, 122.84, 121.88, 111.46, 100.01, 81.05, 71.89, 70.97, 65.29, 57.37, 48.44, 41.96, 36.66, 27.98, 25.00, 19.68; IR (KBr, cm^−1^) *ν*_max_ = 1465, 1501, 1532, 1615, 1735, 2900, 3015, 3270; [Anal. Calcd. for C_33_H_28_Cl_2_N_2_O_2_S: C, 67.46; H, 4.80; N, 4.77; Found: C, 67.35; H, 4.93; N, 4.86]; LC/MS (ESI, *m/z*): 587.20 [M^+^].

*(3S)-7′-(Benzo[b]thiophen-2-yl)-5-chloro-6′-(4-fluorobenzoyl)-3′,6′,7′,7a’-tetrahydro-1′H-spiro[indoline-3,5′-pyrrolo[1,2-c]thiazol]-2-one* (**IIg**). Analog **IIg** was prepared using **2c** (282 mg), 5-chloro-isatin (**3b**) (181 mg), and thioproline (**4a**) (133 mg). Yield: 491 mg (0.92 mmol, 92%); m.p: 130 °C; ^1^H-NMR (400 MHz, DMSO-*d*_6_) δ: 10.54 (s, 1H, NH), 7.90 (d, *J* = 7.9 Hz, 1H, Ar–H), 7.77 (d, *J* = 7.8 Hz, 1H, Ar–H), 7.58 (s, 1H, Ar–H), 7.54–7.43 (m, 3H, Ar–H), 7.39–7.28 (m, 2H, Ar–H), 7.26–7.13 (m, 3H, Ar–H), 6.56 (d, *J* = 8.2 Hz, 1H, Ar–H), 4.77 (d, *J* = 10.9 Hz, 1H, CHCO), 4.30–4.18 (m, 2H, CH_2_), 3.74 (d, *J* = 10.4 Hz, 1H, CH), 3.40 (s, 1H, CH), 3.30–3.22 (m, 1H, CH_2_), 3.15 (s, 1H, CH_2_); ^13^C-NMR (126 MHz, DMSO-*d*_6_) δ: 194.92, 178.15, 166.73, 164.72, 143.14, 141.49, 140.00, 138.81, 133.36, 131.26, 131.18, 130.51, 128.42, 125.73, 125.09, 125.05, 124.80, 123.87, 123.08, 122.93, 116.34, 116.17, 111.63, 74.32, 74.25, 62.38, 54.28, 47.12, 36.54; [Anal. Calcd. for C_28_H_20_ClFN_2_O_2_S_2_: C, 62.85; H, 3.77; N, 5.24; Found: C, 63.04; H, 3.63; N, 5.11]; LC/MS (ESI, *m/z*): 535.10 [M^+^].

*(3S)-1′-(Benzo[b]thiophen-2-yl)-5-chloro-2′-(4-fluorobenzoyl)-1′,2′,5′,5a’,6′,7′,8′,9′,9a’,9b’-decahydrospiro[indoline-3,3′-pyrrolo[2,1-a]isoindol]-2-one* (**IIh**). Analog **IIh** was prepared using **2c** (282 mg), 5-chloro-isatin (**3b**) (181 mg), and (2*S*,3a*S*,7a*S*)-octahydro-1*H*-indole-2-carboxylic acid (**4b**) (169 mg). Yield: 496 mg (0.87 mmol, 87%); m.p: 125 °C; ^1^H-NMR (400 MHz, DMSO-*d*_6_) δ: 10.28 (s, 1H, NH), 7.87 (d, *J* = 7.8 Hz, 1H, Ar–H), 7.74 (d, *J* = 7.6 Hz, 1H, Ar–H), 7.49 (dd, *J* = 8.7, 5.3 Hz, 2H, Ar–H), 7.46 (s, 1H, Ar–H), 7.39 (s, 1H, Ar–H), 7.29 (dd, *J* = 11.6, 7.4 Hz, 2H, Ar–H), 7.20 (t, *J* = 8.7 Hz, 3H, Ar–H), 6.51 (d, *J* = 8.3 Hz, 1H, Ar–H), 4.92 (d, *J* = 11.6 Hz, 1H, CHCO), 4.32 (t, *J* = 10.7 Hz, 1H, CH), 4.22 (t, *J* = 8.8 Hz, 1H, CH), 3.16 (d, *J* = 3.8 Hz, 1H, CH), 2.10 (td, *J* = 11.5, 10.7, 6.6 Hz, 2H, CH_2_), 1.70 (dd, *J* = 11.2, 6.1 Hz, 1H, CH_2_), 1.58–1.43 (m, 2H, CH_2_), 1.39–1.24 (m, 2H, CH_2_), 1.03–0.93 (m, 1H, CH_2_), 0.87 (dd, *J* = 7.5, 4.3 Hz, 1H, CH_2_), 0.79 (t, *J* = 3.3 Hz, 1H, CH_2_), 0.71 (d, *J* = 13.6 Hz, 1H, CH_2_); ^13^C-NMR (101 MHz, DMSO-*d*_6_) δ: 195.22, 180.01, 166.92, 164.40, 143.90, 141.17, 140.10, 138.77, 133.73, 131.21, 129.87, 128.28, 125.87, 124.96, 124.51, 123.70, 122.89, 121.85, 116.17, 111.42, 71.92, 71.01, 70.87, 65.41, 57.37, 48.52, 41.98, 36.70, 28.01, 24.99, 19.69; IR (KBr, cm^−1^) *ν*_max_ = 1485, 1545, 1615, 1715, 2920, 3115, 3275; [Anal. Calcd. for C_33_H_28_ClFN_2_O_2_S: C, 69.40; H, 4.94; N, 4.91; Found: C, 69.61; H, 5.12; N, 4.79]; LC/MS (ESI, *m/z*): 571.20 [M^+^].

*(3S)-7′-(Benzo[b]thiophen-2-yl)-5-chloro-6′-(4-(trifluoromethyl)benzoyl)-3′,6′,7′,7a’-tetrahydro-1′H-spiro[indoline-3,5′-pyrrolo[1,2-c]thiazol]-2-one* (**IIi**). Analog **IIi** was prepared using **2d** (332 mg), 5-chloro-isatin (**3b**) (181 mg), and thioproline (**4a**) (133 mg). Yield: 531 mg (0.91 mmol, 91%); m.p: 114 °C; ^1^H-NMR (400 MHz, DMSO-*d*_6_) δ: 10.52 (s, 1H, NH), 7.91 (d, *J* = 7.6 Hz, 1H, Ar–H), 7.77 (d, *J* = 7.8 Hz, 1H, Ar–H), 7.73 (d, *J* = 8.1 Hz, 2H, Ar–H), 7.61 (s, 1H, Ar–H), 7.56 (d, *J* = 8.1 Hz, 2H, Ar–H), 7.49 (d, *J* = 1.6 Hz, 1H, Ar–H), 7.33 (dt, *J* = 17.7, 7.1 Hz, 2H, Ar–H), 7.26–7.21 (m, 1H, Ar–H), 6.52 (d, *J* = 8.7 Hz, 1H, Ar–H), 4.84 (d, *J* = 10.7 Hz, 1H, CHCO), 4.25 (*q*, *J* = 9.5, 8.0 Hz, 2H, CH_2_), 3.74 (d, *J* = 10.8 Hz, 1H, CH), 3.37 (d, *J* = 10.7 Hz, 1H, CH), 3.27 (d, *J* = 11.5 Hz, 1H, CH_2_), 3.18 (dd, *J* = 11.5, 5.9 Hz, 1H, CH_2_); ^13^C-NMR (101 MHz, DMSO-*d*_6_) δ: 196.30, 177.99, 143.03, 141.56, 140.05, 139.79, 138.87, 133.61, 133.30, 130.72, 128.96, 128.39, 126.18, 125.87, 125.47, 125.16, 124.96, 124.88, 123.95, 123.22, 122.99, 122.76, 111.73, 74.41, 74.13, 62.85, 54.28, 46.97, 36.57; IR (KBr, cm^−1^) *ν*_max_ = 1475, 1534, 1599, 1732, 2998, 3100, 3265; [Anal. Calcd. for C_29_H_20_ClF_3_N_2_O_2_S_2_: C, 59.53; H, 3.45; N, 4.79; Found: C, 59.41; H, 3.55; N, 4.92]; LC/MS (ESI, *m/z*): 585.20 [M^+^].

*(3S)-7′-(Benzo[b]thiophen-2-yl)-6′-(4-(trifluoromethyl)benzoyl)-3′,6′,7′,7a’-tetrahydro-1′H-spiro[indoline-3,5′-pyrrolo[1,2-c]thiazol]-2-one* (**IIj**). Analog **IIj** was obtained using **2d** (332 mg), isatin (**3a**) (147 mg), and thioproline (**4a**) (133 mg). Yield: 456 mg (0.83 mmol, 83%); m.p: 96 °C; ^1^H-NMR (400 MHz, DMSO-*d*_6_) δ: 10.37 (s, 1H, NH), 7.99–7.87 (m, 1H, Ar–H), 7.78 (d, *J* = 7.9 Hz, 1H, Ar–H), 7.69 (d, *J* = 8.1 Hz, 2H Ar–H), 7.58 (s, 1H Ar–H), 7.49 (d, *J* = 8.0 Hz, 2H, Ar–H), 7.39 (d, *J* = 7.6 Hz, 1H, Ar–H), 7.33 (t, *J* = 9.3 Hz, 2H, Ar–H), 7.15 (t, *J* = 7.8 Hz, 1H, Ar–H), 6.97 (t, *J* = 7.7 Hz, 1H, Ar–H), 6.49 (d, *J* = 7.8 Hz, 1H, Ar–H), 4.82 (d, *J* = 10.5 Hz, 1H, CHCO), 4.36–4.18 (m, 2H, CH_2_), 3.73 (d, *J* = 10.3 Hz, 1H, CH), 3.38 (d, *J* = 10.3 Hz, 1H, CH), 3.20 (d, *J* = 5.3 Hz, 2H, CH_2_); ^13^C-NMR (101 MHz, DMSO-*d*_6_) δ: 196.41, 178.40, 143.35, 142.63, 140.08, 139.99, 138.85, 133.34, 133.03, 130.78, 128.83, 128.41, 126.00, 125.50, 125.15, 124.86, 123.94, 123.01, 122.79, 121.82, 110.28, 74.42, 73.81, 63.08, 54.07, 46.88, 36.56; IR (KBr, cm^−1^) *ν*_max_ = 1455, 1550, 1608, 1701, 2915, 3085, 3265; [Anal. Calcd. for C_29_H_21_F_3_N_2_O_2_S_2_: C, 63.26; H, 3.84; N, 5.09; Found: C, 63.15; H, 4.09; N, 5.23]; LC/MS (ESI, *m/z*): 551.20 [M^+^].

*(3S)-1′-(Benzo[b]thiophen-2-yl)-5-chloro-2′-(4-(trifluoromethyl)benzoyl)-1′,2′,5′,5a’,6′,7′,8′,9′,9a’,9b’-decahydrospiro[indoline-3,3′-pyrrolo[2,1-a]isoindol]-2-one* (**IIk**). Analog **IIk** was prepared using **2d** (332 mg), 5-chloro-isatin (**3b**) (181 mg), and (2*S*,3a*S*,7a*S*)-octahydro-1*H*-indole-2-carboxylic acid (**4b**) (169 mg). Yield: 527 mg (0.85 mmol, 85%); m.p: 134 °C; ^1^H-NMR (400 MHz, DMSO-*d*_6_) δ: 10.27 (s, 1H, NH), 7.88 (d, *J* = 7.9 Hz, 1H, Ar–H), 7.74 (dd, *J* = 8.1, 3.6 Hz, 3H, Ar–H), 7.56 (d, *J* = 8.0 Hz, 2H, Ar–H), 7.49 (s, 1H, Ar–H), 7.43 (s, 1H, Ar–H), 7.35–7.24 (m, 2H, Ar–H), 7.20 (d, *J* = 9.1 Hz, 1H, Ar–H), 6.47 (d, *J* = 8.3 Hz, 1H, Ar–H), 4.99 (d, *J* = 11.7 Hz, 1H, CHCO), 4.41–4.30 (m, 1H, CH), 4.23 (t, *J* = 7.9 Hz, 1H, CH), 3.17 (d, *J* = 3.7 Hz, 1H, CH), 2.12 (t, *J* = 8.3 Hz, 2H, CH_2_), 1.71 (dd, *J* = 10.6, 6.0 Hz, 1H, CH_2_), 1.51 (s, 2H, CH_2_), 1.32 (t, *J* = 12.4 Hz, 2H, CH_2_), 1.09 (d, *J* = 12.4 Hz, 1H, CH_2_), 1.00 (t, *J* = 12.5 Hz, 1H, CH_2_), 0.89 (t, *J* = 13.7 Hz, 1H, CH_2_), 0.70 (d, *J* = 13.7 Hz, 1H, CH_2_); ^13^C-NMR (126 MHz, DMSO-*d*_6_) δ: 196.41, 179.79, 143.70, 141.14, 140.07, 138.74, 133.36, 133.11, 130.06, 128.89, 128.21, 126.05, 126.02, 125.95, 125.70, 125.22, 124.92, 124.51, 123.69, 123.05, 122.81, 121.86, 111.44, 71.74, 70.98, 65.60, 57.33, 48.29, 41.85, 36.57, 27.97, 27.90, 24.93, 19.65; IR (KBr, cm^−1^) *ν*_max_ = 1450, 1485, 1535, 1623, 1710, 2905, 3035, 3355; [Anal. Calcd. for C_34_H_28_ClF_3_N_2_O_2_S: C, 65.75; H, 4.54; N, 4.51; Found: C, 65.61; H, 4.63; N, 4.42]; LC/MS (ESI, *m/z*): 621.20 [M^+^].

*(3S)-7′-(Benzo[b]thiophen-2-yl)-6′-(4-bromobenzoyl)-5-chloro-3′,6′,7′,7a’-tetrahydro-1′H-spiro[indoline-3,5′-pyrrolo[1,2-c]thiazol]-2-one* (**IIl**). Analog **IIl** was obtained using **2e** (341 mg), 5-chloro-isatin (**3b**) (181 mg), and thioproline (**4a**) (133 mg). Yield 545 mg (0.92 mmol, 92%); m.p:108 °C; ^1^H-NMR (400 MHz, DMSO-*d*_6_) δ: 10.55 (s, 1H, NH), 7.91 (d, *J* = 7.9 Hz, 1H, Ar–H), 7.77 (d, *J* = 7.9 Hz, 1H, Ar–H), 7.60–7.46 (m, 4H, Ar–H), 7.33 (*q*, *J* = 5.5 Hz, 3H, Ar–H), 7.28–7.21 (m, 2H, Ar–H), 6.56 (d, *J* = 8.3 Hz, 1H, Ar–H), 4.75 (d, *J* = 11.1 Hz, 1H, CHCO), 4.23 (d, *J* = 11.0 Hz, 2H, CH_2_), 3.74 (d, *J* = 10.7 Hz, 1H, CH), 3.40 (s, 1H, CH), 3.29–3.22 (m, 1H, CH_2_), 3.17 (dd, *J* = 11.3, 5.7 Hz, 1H, CH_2_); ^13^C-NMR (126 MHz, DMSO-*d*_6_) δ: 195.67, 178.03, 143.06, 141.49, 139.99, 138.79, 135.58, 132.27, 130.58, 130.06, 128.56, 128.40, 125.71, 125.13, 124.97, 124.84, 123.91, 123.14, 122.96, 111.68, 74.33, 74.21, 62.37, 54.28, 47.03, 36.52; IR (KBr, cm^−1^) *ν*_max_ = 1490, 1550, 1625, 1725, 2915, 3050, 3250; [Anal. Calcd. for C_28_H_20_BrClN_2_O_2_S_2_: C, 56.43; H, 3.38; N, 4.70; Found: C, 56.33; H, 3.49; N, 4.91]; LC/MS (ESI, *m/z*): 595.20 [M^+^].

*(3S)-7′-(Benzo[b]thiophen-2-yl)-6′-(4-bromobenzoyl)-3′,6′,7′,7a’-tetrahydro-1′H-spiro[indoline-3,5′-pyrrolo[1,2-c]thiazol]-2-one* (**IIm**). Analog **IIm** was obtained using **2e** (341 mg), isatin (**3a**) (147 mg), and thioproline (**4a**) (133 mg). Yield: 492 mg (0.88 mmol, 88%); m.p:100 °C; ^1^H-NMR (400 MHz, DMSO-*d*_6_) δ: 10.41 (s, 1H, NH), 7.91 (d, *J* = 7.8 Hz, 1H, Ar–H), 7.77 (d, *J* = 7.6 Hz, 1H, Ar–H), 7.54 (t, *J* = 4.4 Hz, 3H, Ar–H), 7.33 (ddd, *J* = 29.2, 17.6, 8.3 Hz, 5H, Ar–H), 7.15 (s, 1H, Ar–H), 6.96 (s, 1H, Ar–H), 6.53 (d, *J* = 7.9 Hz, 1H, Ar–H), 4.73 (d, *J* = 10.7 Hz, 1H, CHCO), 4.33–4.18 (m, 2H, CH_2_), 3.72 (d, *J* = 10.3 Hz, 1H, CH), 3.37 (s, 1H, CH), 3.19 (d, *J* = 4.8 Hz, 2H, CH_2_); ^13^C-NMR (126 MHz, DMSO-*d*_6_) δ 195.76, 178.45, 143.40, 142.55, 140.02, 138.78, 135.76, 132.09, 130.62, 129.97, 128.44, 128.21, 125.10, 124.79, 123.88, 123.03, 122.94, 122.89, 121.70, 110.23, 74.33, 73.89, 62.55, 54.05, 46.95, 36.50; IR (KBr, cm^−1^) *ν*_max_ = 1480, 1510, 1608, 1720, 2910, 3055, 3245; [Anal. Calcd. for C_28_H_21_BrN_2_O_2_S_2_: C, 59.89; H, 3.77; N, 4.99; Found: C, 60.03; H, 3.65; N, 5.08]; LC/MS (ESI, *m/z*): 561.20 [M^+^].

*(3S)-1′-(Benzo[b]thiophen-2-yl)-2′-(4-bromobenzoyl)-5-chloro-1′,2′,5′,5a’,6′,7′,8′,9′,9a’,9b’-decahydrospiro[indoline-3,3′-pyrrolo[2,1-a]isoindol]-2-one* (**IIn**). Analog **IIn** was prepared using **2e** (341 mg), 5-chloro-isatin (**3b**) (181 mg), and (2*S*,3a*S*,7a*S*)-octahydro-1*H*-indole-2-carboxylic acid (**4b**) (169 mg) in equimolar amounts. Yield: 567 mg (0.9 mmol, 90%); m.p:136 °C; ^1^H-NMR (400 MHz, DMSO-*d*_6_) δ: 10.31 (s, 1H, NH), 7.87 (d, *J* = 7.9 Hz, 1H, Ar–H), 7.74 (d, *J* = 7.6 Hz, 1H, Ar–H), 7.58 (d, *J* = 8.2 Hz, 2H, Ar–H), 7.46 (s, 1H, Ar–H), 7.39 (s, 1H, Ar–H), 7.30 (dd, *J* = 19.2, 7.8 Hz, 4H, Ar–H), 7.19 (d, *J* = 8.6 Hz, 1H, Ar–H), 6.52 (d, *J* = 8.2 Hz, 1H, Ar–H), 4.90 (d, *J* = 11.7 Hz, 1H, CHCO), 4.32 (t, *J* = 10.8 Hz, 1H, CH), 4.22 (t, *J* = 8.8 Hz, 1H, CH), 3.16 (d, *J* = 3.9 Hz, 1H, CH), 2.11 (dd, *J* = 9.9, 4.2 Hz, 2H, CH_2_), 1.70 (dd, *J* = 11.0, 6.0 Hz, 1H, CH_2_), 1.49 (d, *J* = 12.6 Hz, 2H, CH_2_), 1.32 (t, *J* = 11.2 Hz, 2H, CH_2_), 1.09 (d, *J* = 12.6 Hz, 1H, CH_2_), 0.98 (d, *J* = 12.9 Hz, 1H, CH_2_), 0.86 (d, *J* = 13.2 Hz, 1H, CH_2_), 0.70 (d, *J* = 13.7 Hz, 1H, CH_2_); ^13^C-NMR (126 MHz, DMSO-*d*_6_) δ: 195.86, 179.87, 143.79, 141.13, 140.04, 138.72, 135.91, 132.14, 130.06, 129.96, 128.25, 125.85, 125.77, 124.92, 124.49, 123.68, 122.81, 121.83, 111.43, 71.84, 70.92, 65.26, 57.33, 48.39, 41.86, 36.61, 27.98, 27.90, 24.94, 19.65; IR (KBr, cm^−1^) *ν*_max_ = 1495, 1615, 1712, 2920, 3245; [Anal. Calcd. for C_33_H_28_BrClN_2_O_2_S: C, 62.71; H, 4.47; N, 4.43; Found: C, 62.85; H, 4.56; N, 4.53]; LC/MS (ESI, *m/z*): 633.20 [M^+^].

## 4. Conclusions

A series of novel spiro-heterocycles incorporating the benzo[*b*]thiophene motif were prepared and their AChE inhibitory activity was evaluated. The results revealed that among the studied compounds, analog **IIc** was the most active AChE inhibitor with an IC_50_ value of 20,840 µM L^−1^. Molecular docking studies were also performed to elucidate the structural features and interactions responsible for the inhibitory potential of these compounds against the target protein. The docking results further confirmed the experimental findings and provided significant information on the binding mechanism of the novel analogues to the AChE enzyme.

## Figures and Tables

**Table 1:** Chemical structures of the synthesized spiro-oxindole analogs **IIa**–**n** and their AChE inhibitory activity.

#	Chemical Structures	AChE Inhibition IC_50_ (µM/L)
**1**	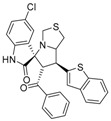 **IIa**	85,560
**2**	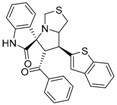 **IIb**	88,410
**3**	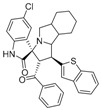 **IIc**	20,840
**4**	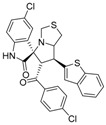 **IId**	37,670
**5**	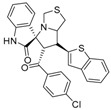 **IIe**	50,590
**6**	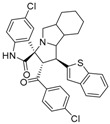 **IIf**	34,020
**7**	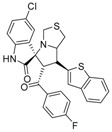 **IIg**	23,040
**8**	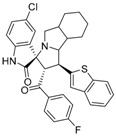 **IIh**	121,690
**9**	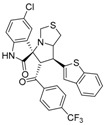 **IIi**	72,380
**10**	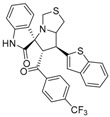 **IIj**	75,980
**11**	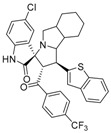 **IIk**	41,530
**12**	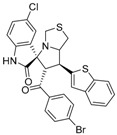 **IIl**	29,760
**13**	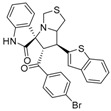 **IIm**	41,450
**14**	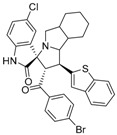 **IIn**	36,830
**STD**	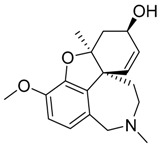 **Galantamine**	**2090** [7,8,9,10,11] **3400** [34] **0.35** [35]

**Table 2:** Docking scores of the selected compound of spiro-benzothiophene series with AChE.

S.No.	Compounds Name	Scores	No. of Hydrogen Bonds
1	**IIc**	−6.54	3
2	**IIf**	−6.01	3
3	**IIg**	−6.23	3
4	**IIl**	−6.19	4
5	**Galantamine**	−9.28	2

## Supplementary Materials

The following are available online: experimental protocol for the AChE assay; Copies of the spectrum; and IC_50_ diagram of the AChE assay.
