# Supplementary material for: Synthesis of a New Class of Spirooxindole–Benzo[b]Thiophene-Based Molecules as Acetylcholinesterase Inhibitors
Source: Molecules. 2020 Oct 13;25(20):4671. doi: 10.3390/molecules25204671 (PMC7594047; doi:10.3390/molecules25204671)
Supplement: Supplementary file 1 [file molecules-25-04671-s001.pdf]

SUPPORTING INFORMATION

Synthesis of a New Class of Spirooxindole–Benzo[*b*]Thiophene-Based Molecules as Acetylcholinesterase Inhibitors

Assem Barakat [1,2,*], Saeed Alshahrani [1], Abdullah Mohammed Al-Majid [1], M. Ali [1], Mezna Saleh Altowyan[3], Mohammad Shahidul Islam [1], Abdullah Saleh Alamary [1], and Sajda Ashraf [4] and Zaheer Ul-Haq[4]

[1] Department of Chemistry, College of Science, King Saud University, P. O. Box 2455, Riyadh 11451, Saudi Arabia; chemistry99y@gmail.com (S.A.); amajid@ksu.edu.sa (A.M.A.-M); maly.c@ksu.edu.sa (M.A.); mislam@ksu.edu.sa (M.S.I); alamary1401@yahoo.com (A.S.A.).

[2] Department of Chemistry, Faculty of Science, Alexandria University, P.O. Box 426, Ibrahimia, Alexandria 21321, Egypt.

[3] Department of Chemistry, College of Science, Princess Nourah Bint Abdulrahman University, Riyadh, Saudi Arabia; msaltowyan@pnu.edu.sa (M.S.A).

[4] Dr. Panjwani Center for Molecular medicine and Drug Research, International Center for Chemical and Biological Sciences, University of Karachi, Karachi-75270, Pakistan; sajda.ashraf@yahoo.com (S.A.); zaheer_qasmi@hotmail.com (Z.U.-H.)

* Correspondence: ambarakat@ksu.edu.sa. Tel.: +966-11467-5901; Fax: +966-11467-5992

Table of Content:

1. Acetylcholinesterase (AChE) inhibitory assay.
2. Docking protocol
3. Copies of NMR spectrum.
4. Copies of the IC_{50} for the synthesized compounds.

Acetylcholinesterase (AChE) inhibitory assay [1].

140 μL of 0.1 M sodium phosphate buffer of pH 8 was first added to a 96-wells microplate followed by 20 μL of test samples and 20 μL of 0.09 units/mL acetylcholinesterase enzyme from electric eel (Sigma-Aldrich, CAS Number 9000-81-1). After 15 min of incubation at 25 °C, 10 μL of 10 mM 5,5′-dithiobis-2-nitrobenzoic acid (DTNB, Sigma-Aldrich, CAS Number 69-78-3) was added into each well followed by 10 μL of 14 mM acetylthiocholine iodide (Sigma-Aldrich, CAS Number 1866-15-5). Thirty min after the initiation of enzymatic reaction, absorbance of the colored end-product was measured using StatFax-2100 microplate reader (Awareness Technology, Inc., USA) at 412 nm. Each test was conducted in triplicates. Test samples were prepared in DMSO at an initial concentration of 1 mg/mL (1000 ppm). The concentration of DMSO in final reaction mixture was 1%. At this concentration, DMSO has no inhibitory effect on acetylcholinesterase enzyme. The initial screening was carried out at 50 μg/mL of test samples in 1% DMSO. Absorbance of the test samples was corrected by subtracting the absorbance of their respective blank. Percentage enzyme inhibition is calculated using the following formula

$$\text{Percentage of inhibition} = \frac{\text{Absorbance of control - Absorbance of Sample}}{\text{Absorbance of control}} \text{ X } 100$$

Subsequently, the determination of IC50 was carried out using a set of six concentrations (1.56-50 μg/mL).

Docking protocol

To investigate the binding mechanism of synthesized analogs of spiro-benzothiophene with acetylcholinesterase, molecular docking was carried out through MOE-Dock program to find the potential interactions require for the catalytic activity of AchE. The compound structures were constructed by Builder module in MOE2019 suit [2]. Hydrogen atoms were added and MMFF94x force field [3] was applied to calculate partial charges. Afterward the structures were minimized until gradient was reached to 0.1 rms. The X-ray crystal structure of human AChE complex with galantamine inhibitor retrieved from protein data bank with PDB ID:4EY6 [4] and prepared using protein preparation module in MOE. The protein preparation step included correction of stereochemistry, atom type, deleting of unwanted atoms and non-conserved water molecules. The protein was further optimized by charge and minimization step using Amber99 forcefield in MOE 2019 and saved in pdb format. To test the docking parameters of the software, redocking experiments was carried out. The cognate ligand was extracted and redocked into the binding cavity of the target protein. The reliable RMSD of 0.81 Å (Figure 1) assured the accuracy of the software to continue docking studies of spiro-benzothiophene derivatives. Each molecule was subjected to 100 docking runs using default MOE docking parameters i.e. Triangle Matcher Algorithm with two rescoring functions London dG and

GBVI/WSA dG. Based on docking score, ten lowest energy conformations of each molecule were selected for visual inspection.

References

1. Kumar, R..;, Almansour, A.I.; Arumugam, N.; Althomili, D.M.Q.; Altaf, M.; Basiri, A.; Kotresha, D.; Manohar, T.S.; Venketesh, S. Ionic liquid-enabled synthesis, cholinesterase inhibitory activity, and molecular docking study of highly functionalized tetrasubstituted pyrrolidines. *Bioorg Chem*., **2018,** *77*, 263.
2. Halgren, Thomas A. Merck Molecular Force Field. I. Basis, Form, Scope, Parameterization, and Performance of MMFF94." *J. Comp. Chem.***1996**, *17* (5–6): 490.
3. "Molecular Operating Environment (MOE), 2013.08; Chemical Computing Group ULC, 1010 Sherbooke St. West, Suite #910, Montreal, QC, Canada, H3A 2R7, 2018." 2018. 2018.
4. Cheung, J.; Rudolph, M.J.; Burshteyn, F.; Cassidy, M.S.; Gary, E.N.; Love, J.; Height, J.J. Structures of human acetylcholinesterase in complex with pharmacologically important ligands. *J Med Chem.* **2012,** *55*(22), 10282.

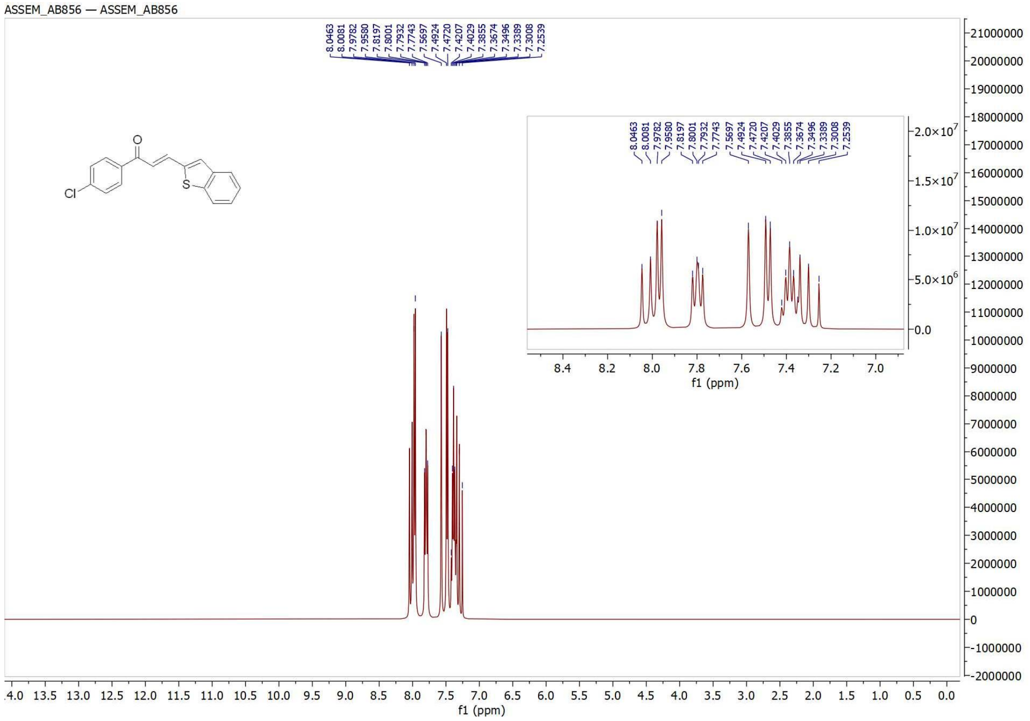

Figure S1: ^{1}HNMR of **2b**

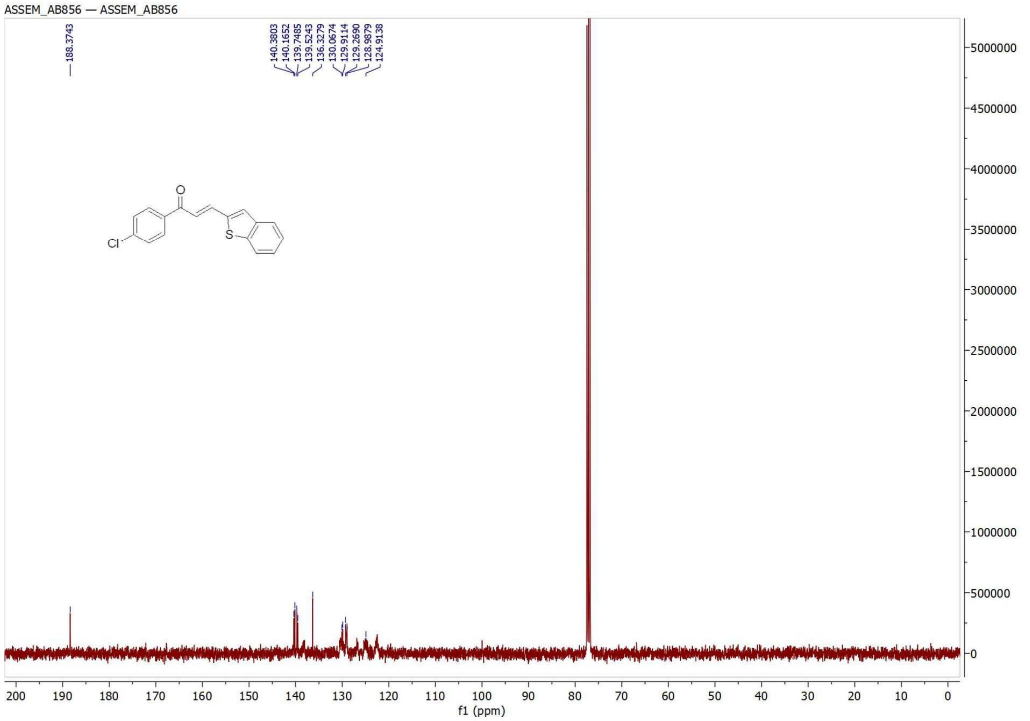

Figure S2: ^{13}CNMR of **2b**

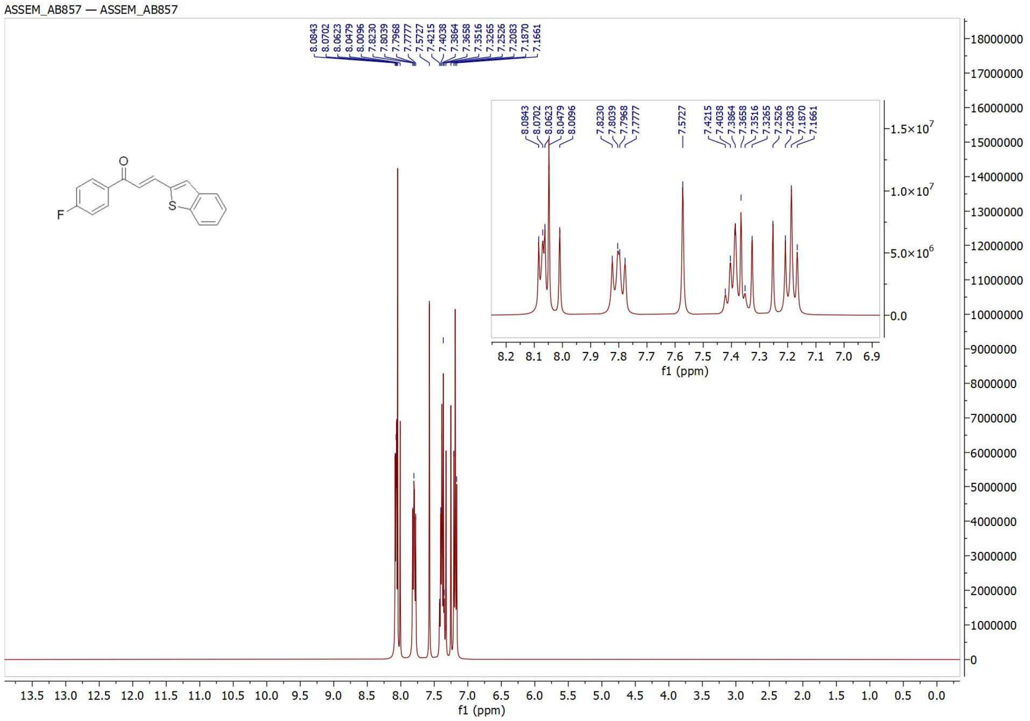

Figure S3: ^{1}HNMR of **2c**

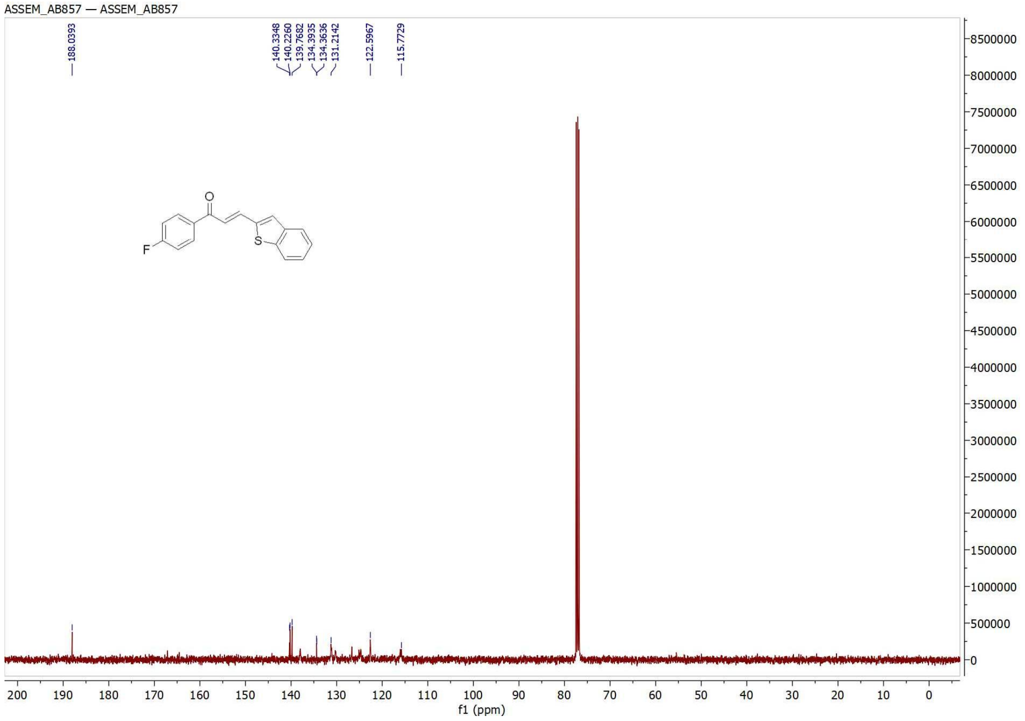

Figure S4: ^{13}CNMR of **2c**

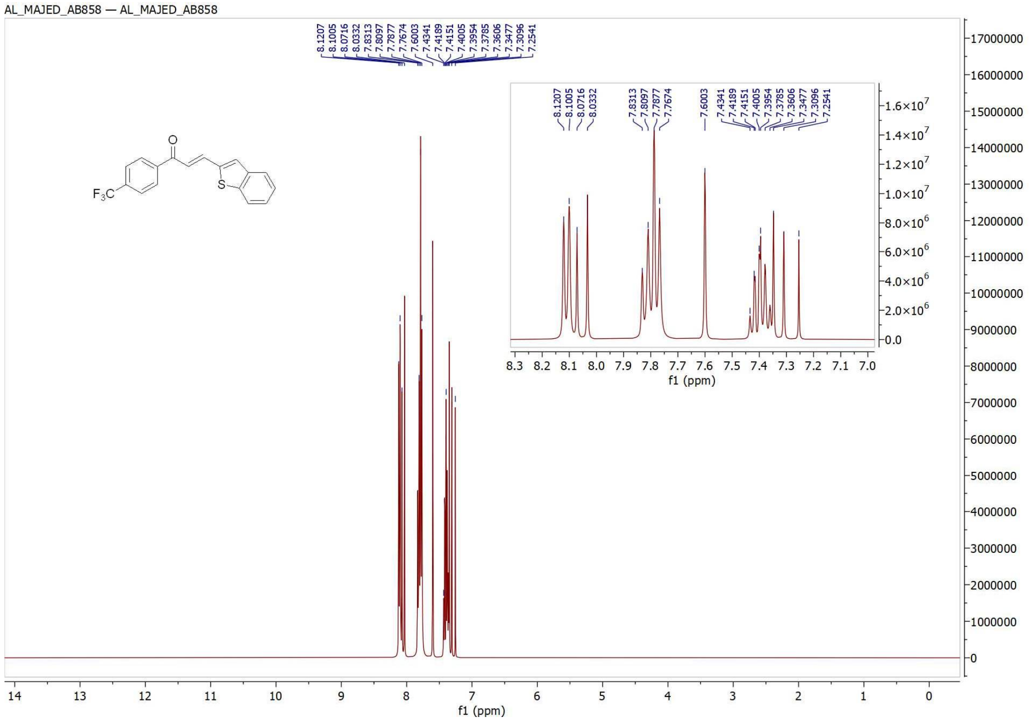

Figure S5: ^{1}HNMR of **2d**

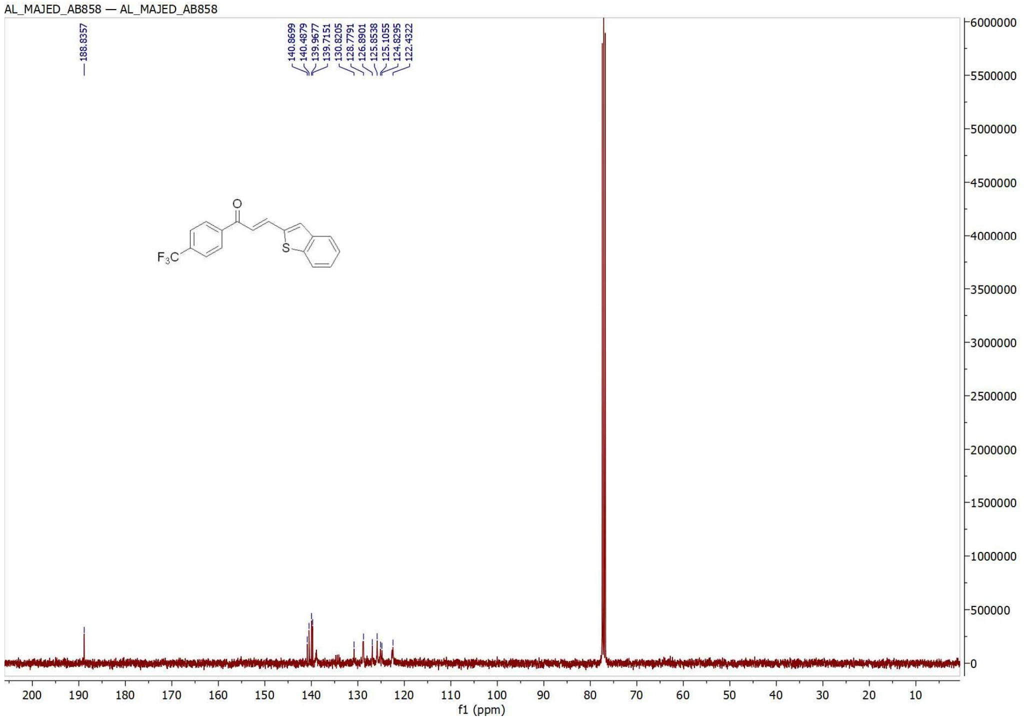

Figure S6: ^{13}CNMR of **2d**

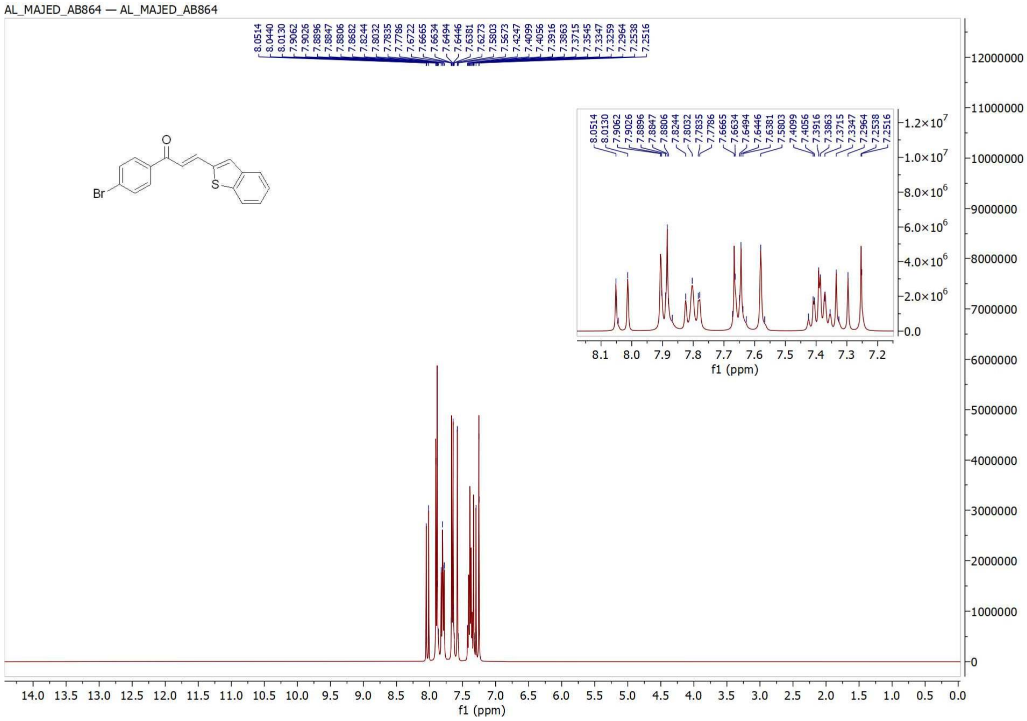

Figure S7: ^{1}HNMR of **2e**

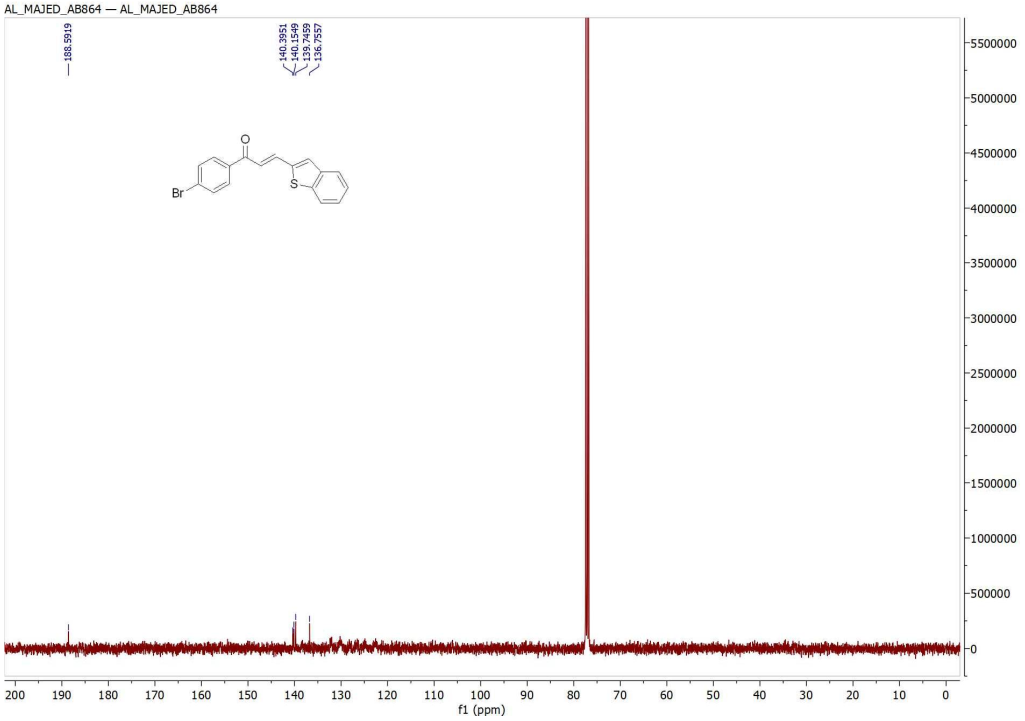

Figure S8: ^{13}CNMR of **2e**

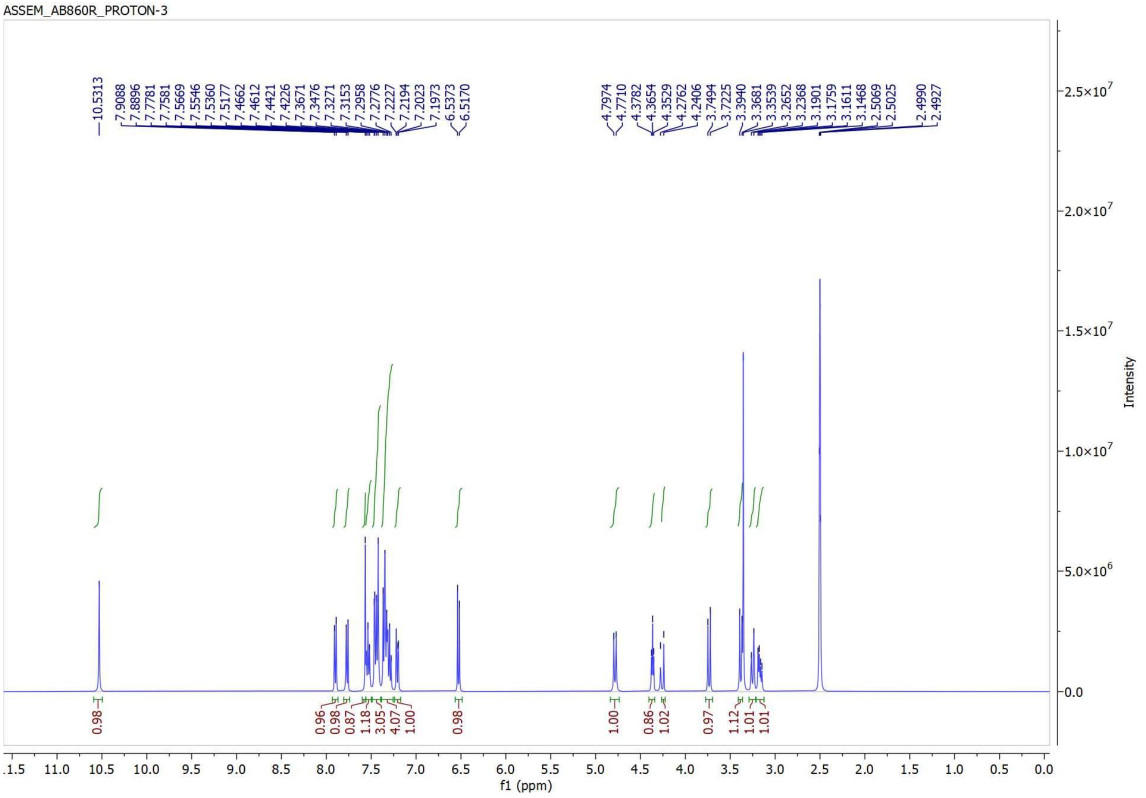

Figure S9: ^{1}HNMR of **IIa**

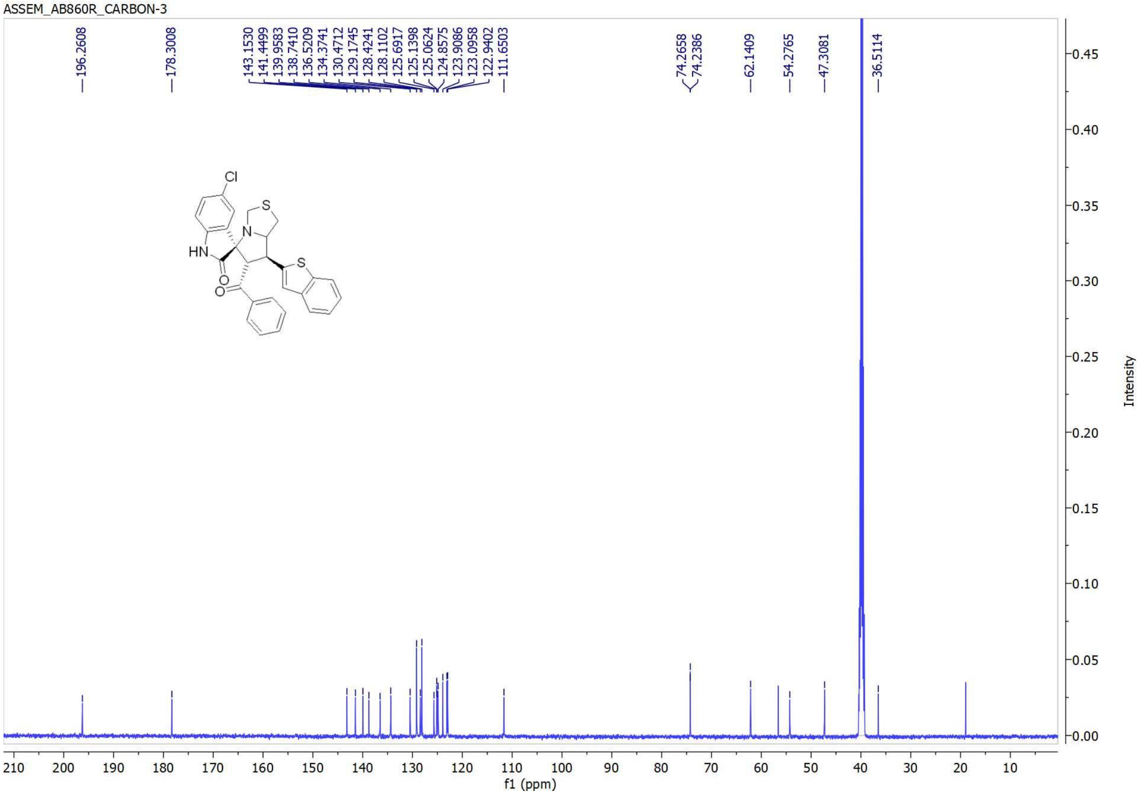

Figure S10: ^{13}CNMR of **IIa**

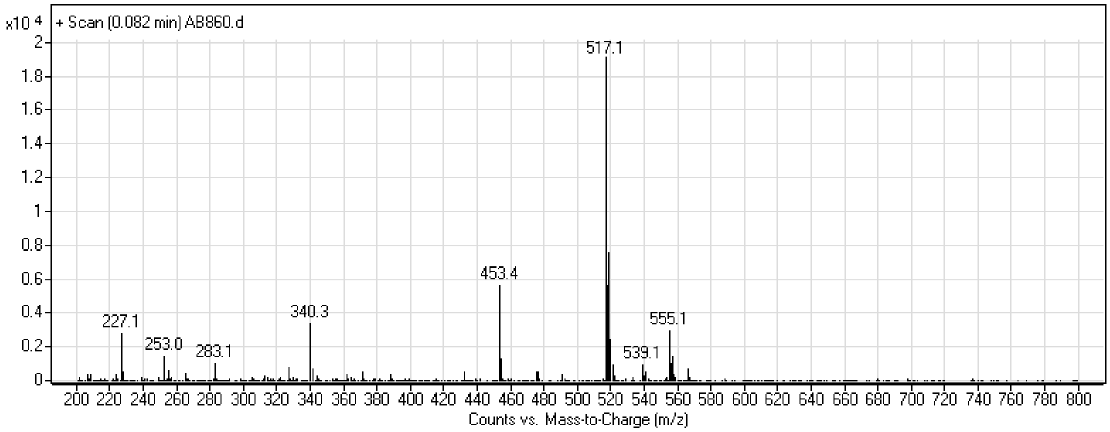

Figure S11: MS of **IIa**

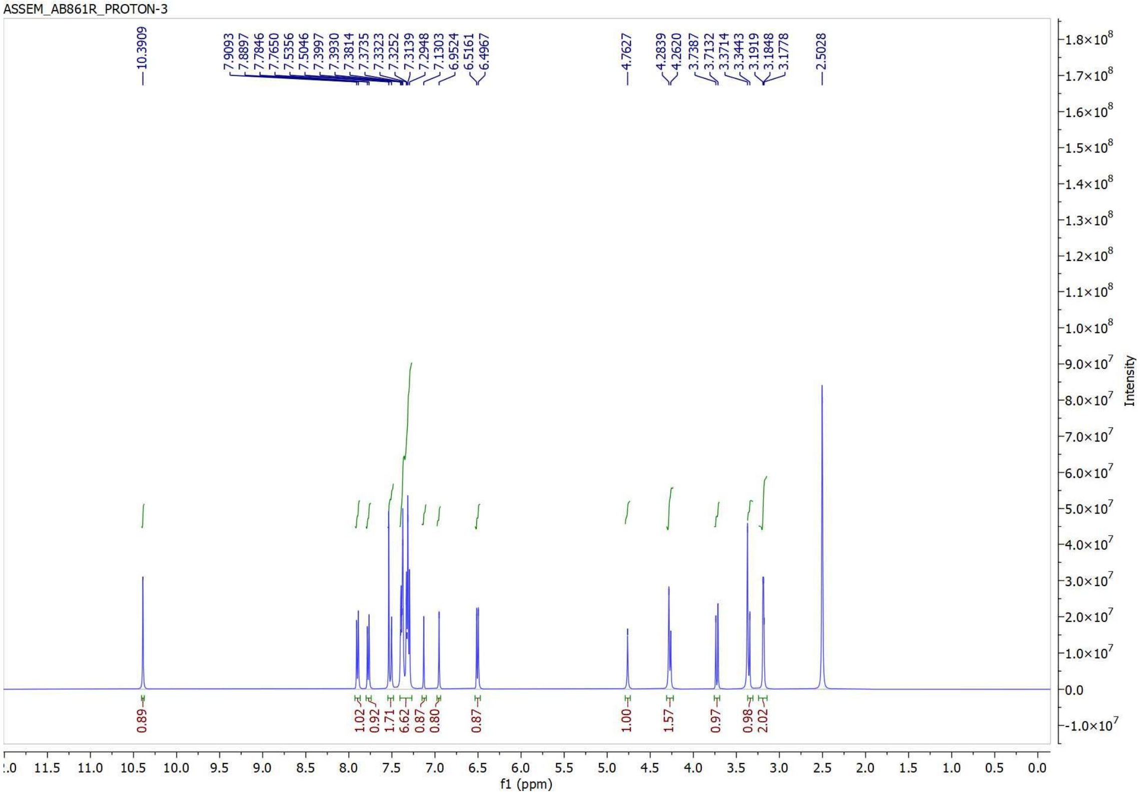

Figure S12: ^{1}HNMR of **IIb**

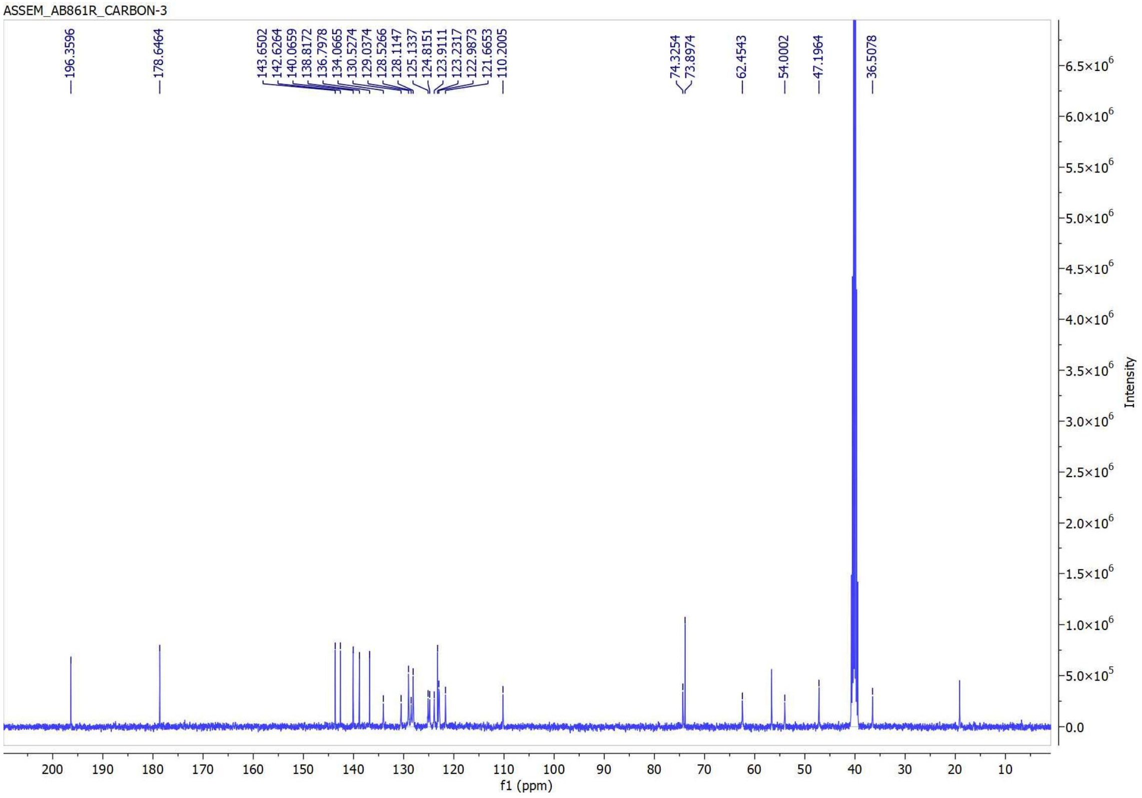

Figure S13: ^{13}CNMR of **IIb**

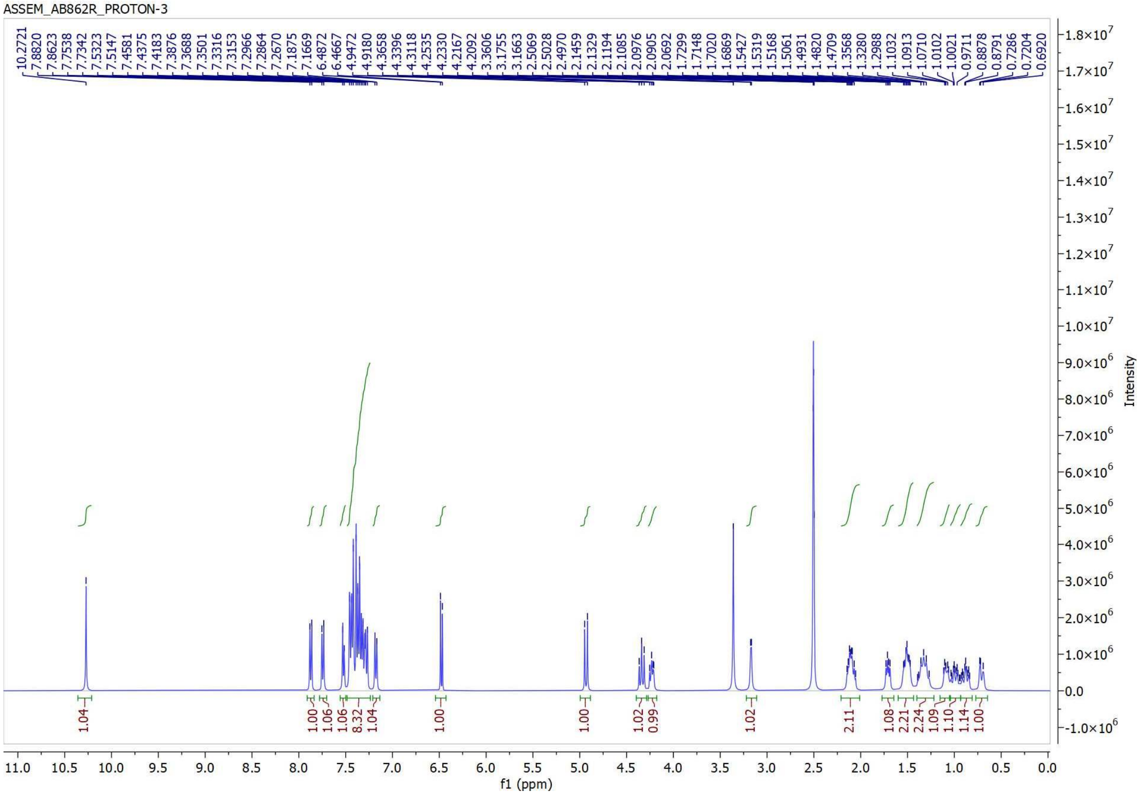

Figure S14: ^{1}HNMR of **IIc**

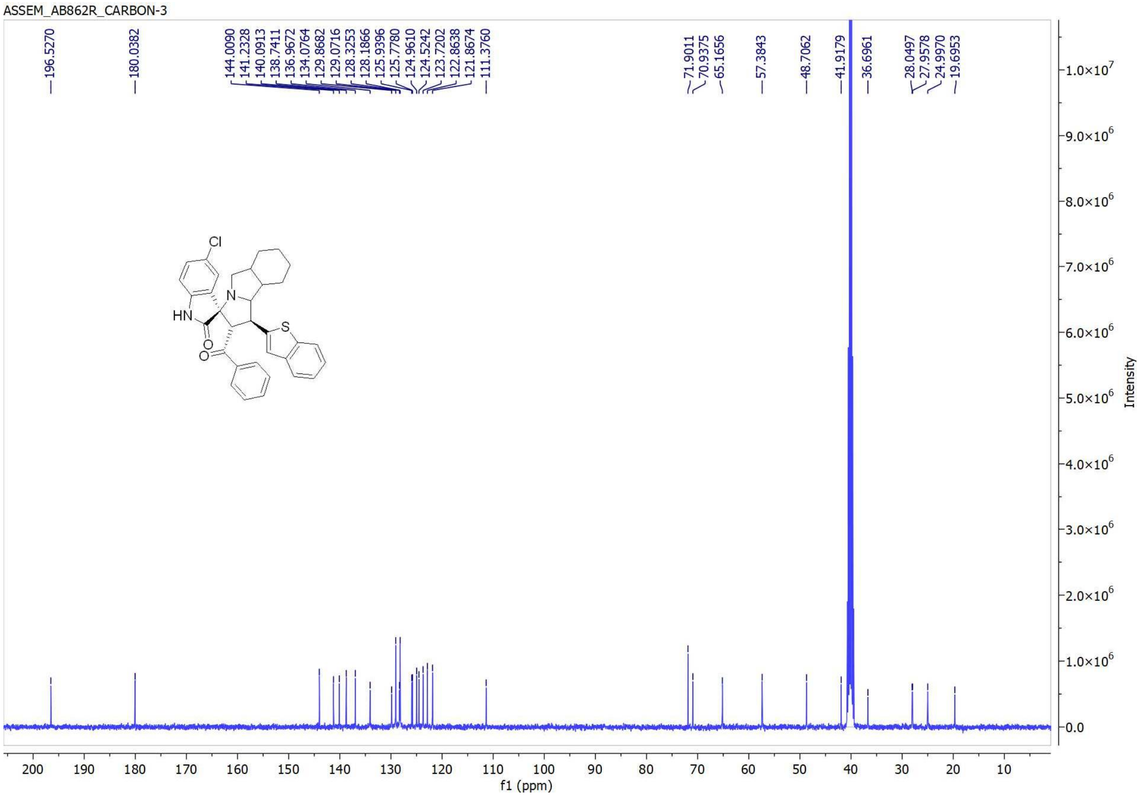

Figure S15: ^{13}CNMR of **IIc**

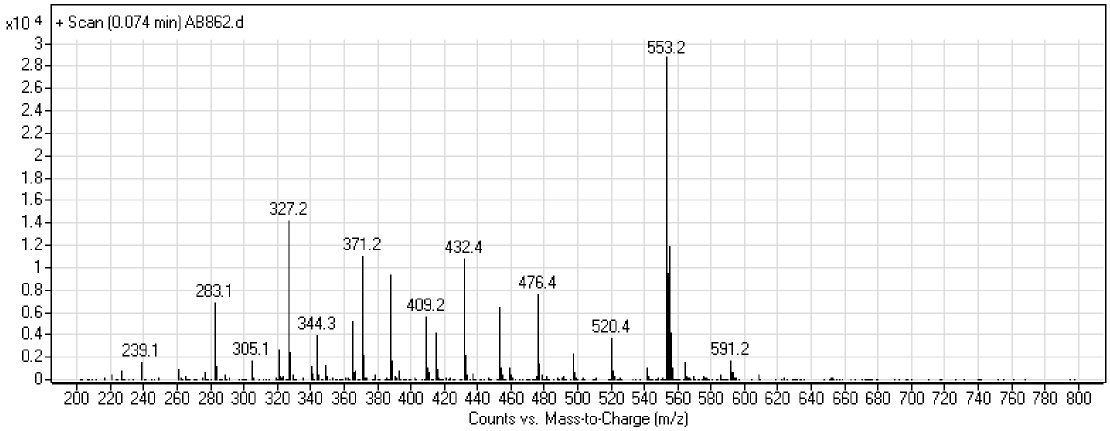

Figure S16: MS of **IIc**

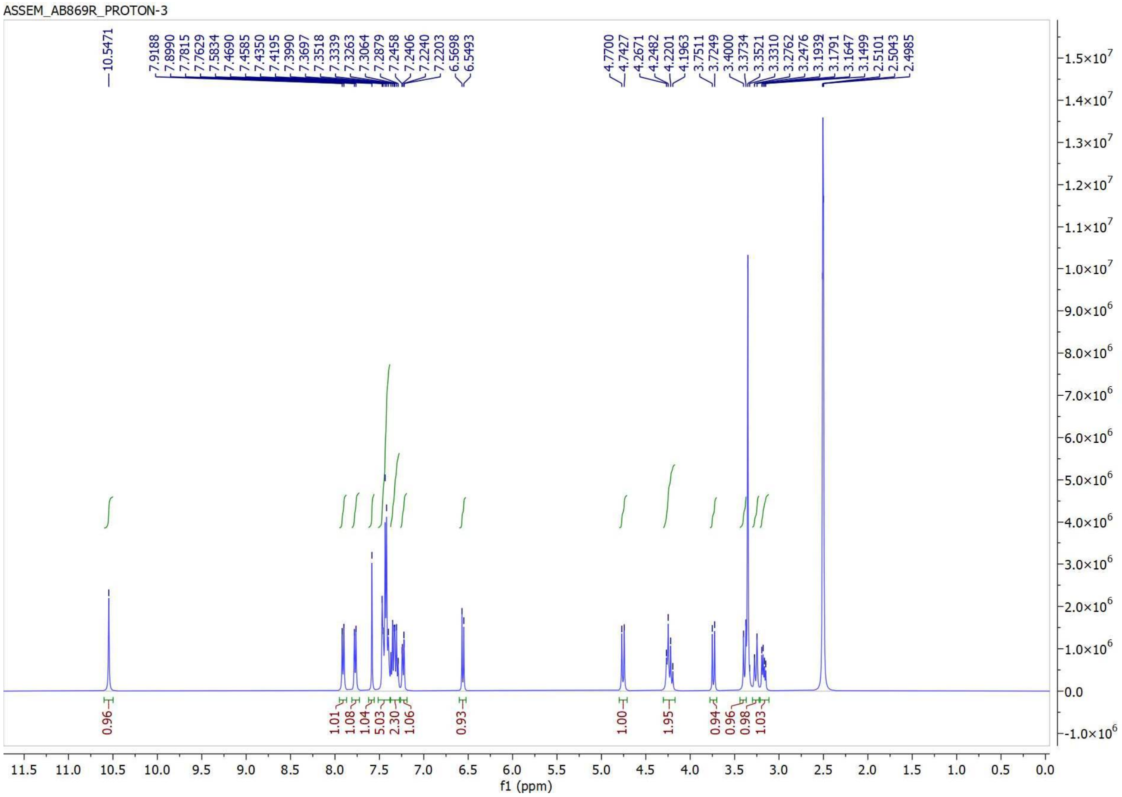

Figure S17: ^{1}HNMR of **IId**

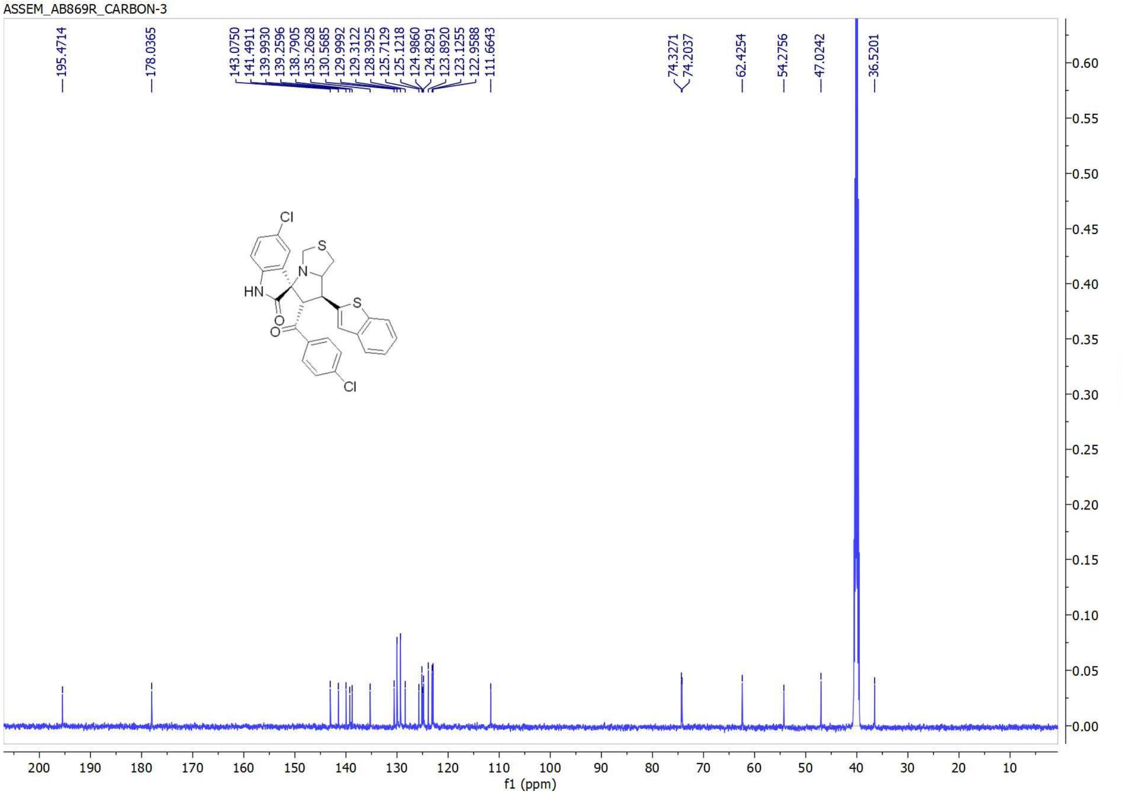

Figure S18: ^{13}CNMR of **IId**

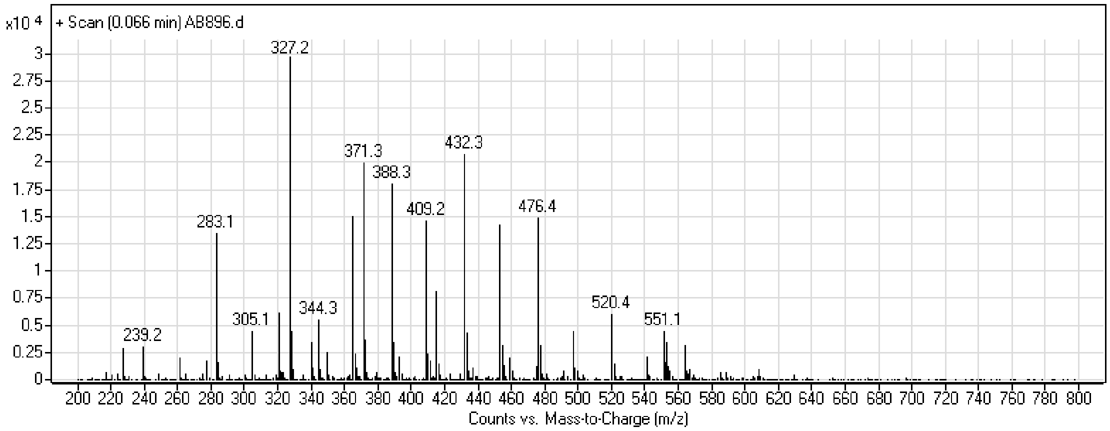

Figure S19: MS of **IId**

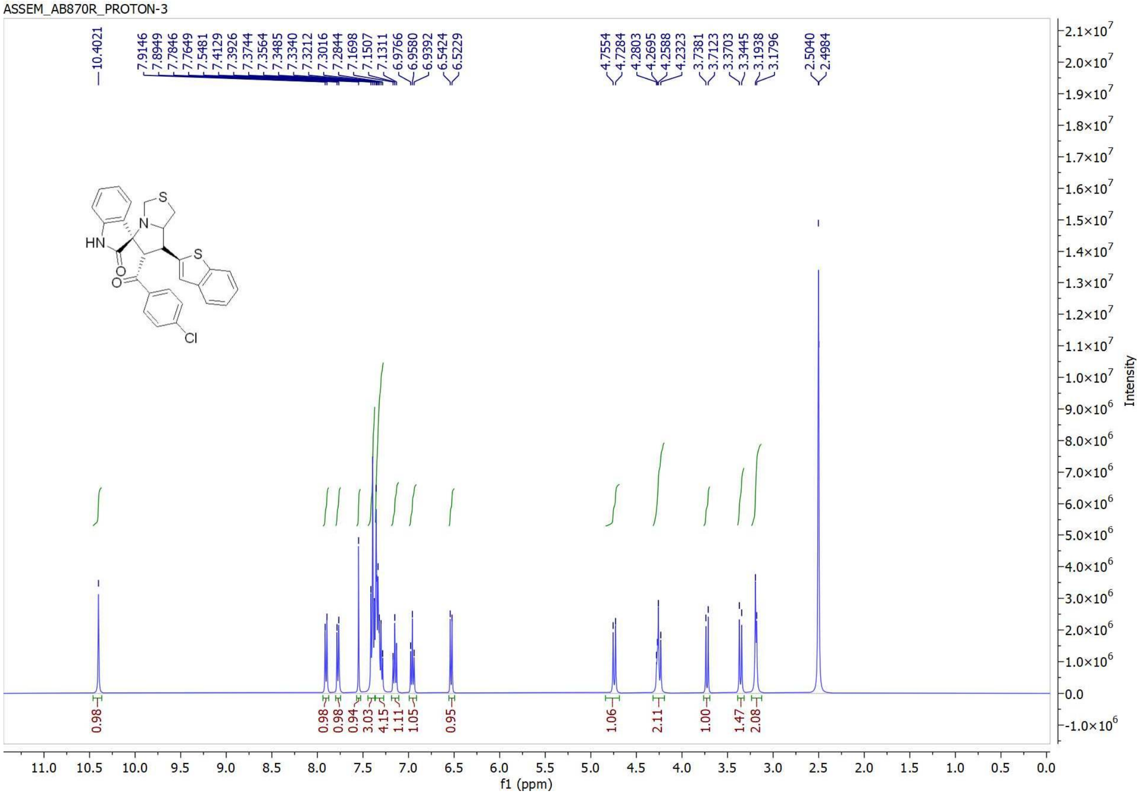

Figure S20: ^{1}HNMR of **IIe**

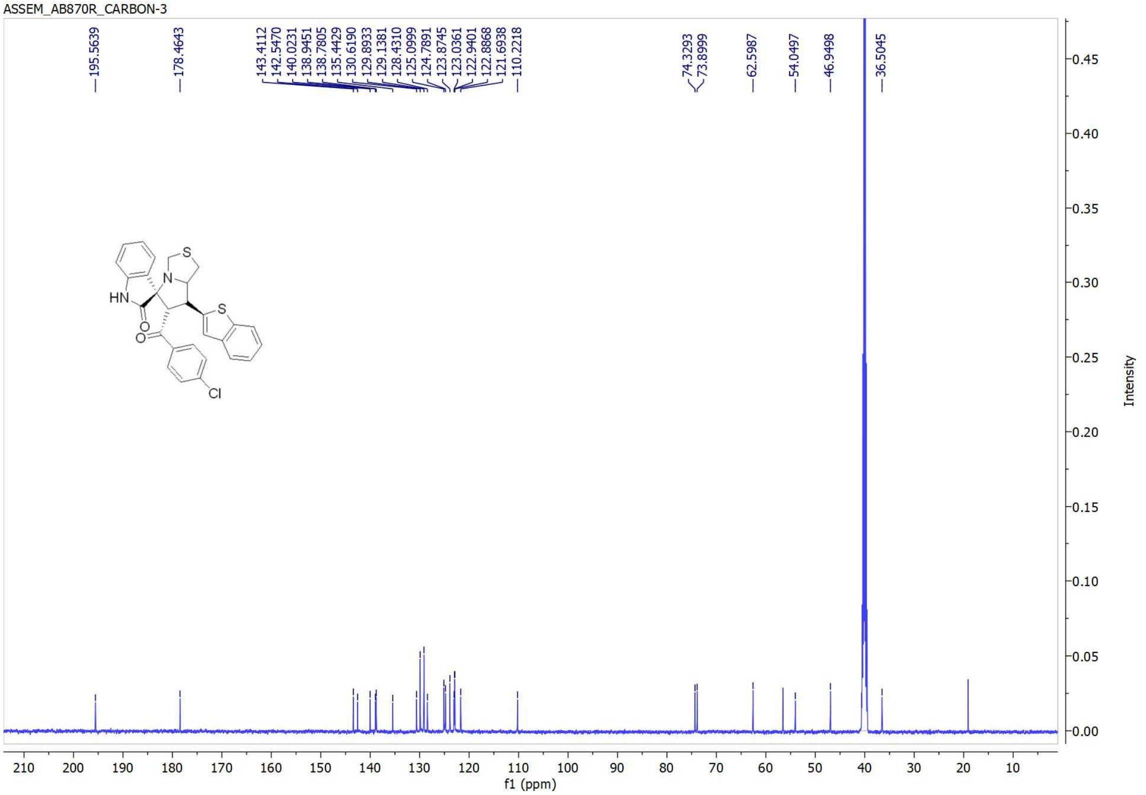

Figure S21: ^{13}CNMR of **IIe**

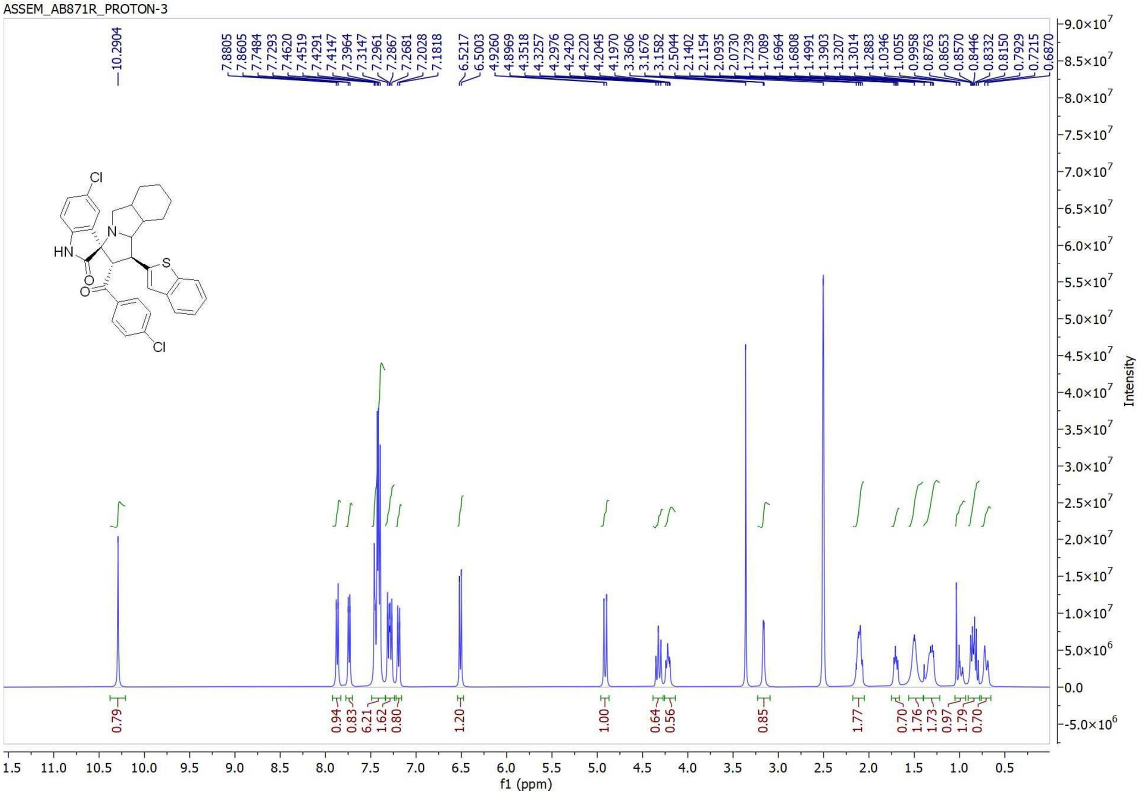

Figure S22: ^{1}HNMR of **IIf**

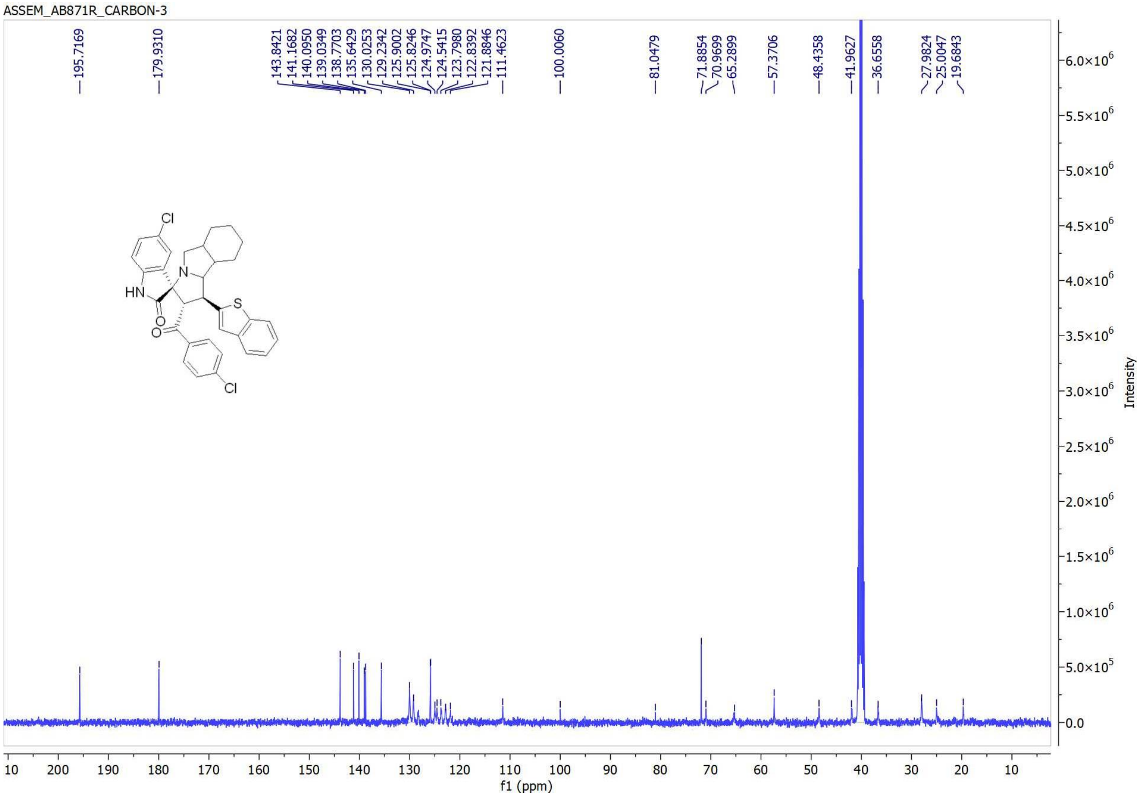

Figure S23: ^{13}CNMR of **IIf**

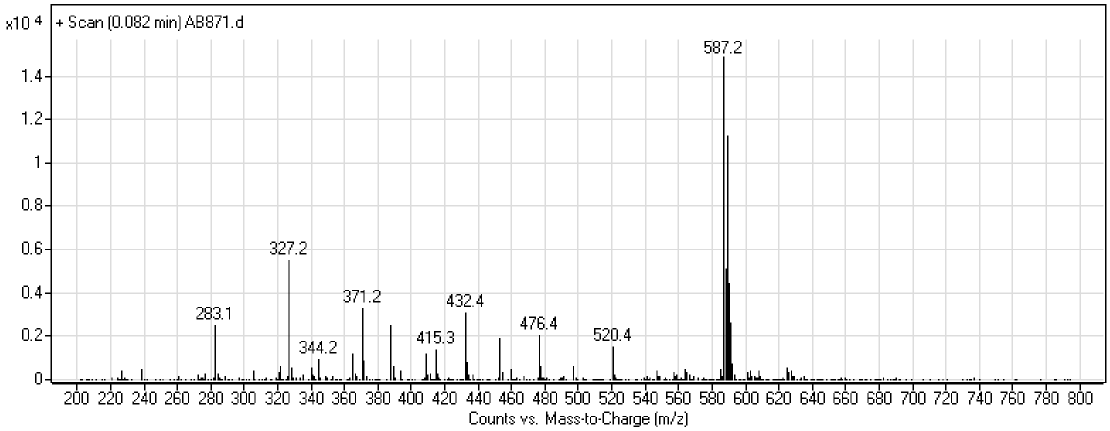

Figure S24: MS of **IIf**

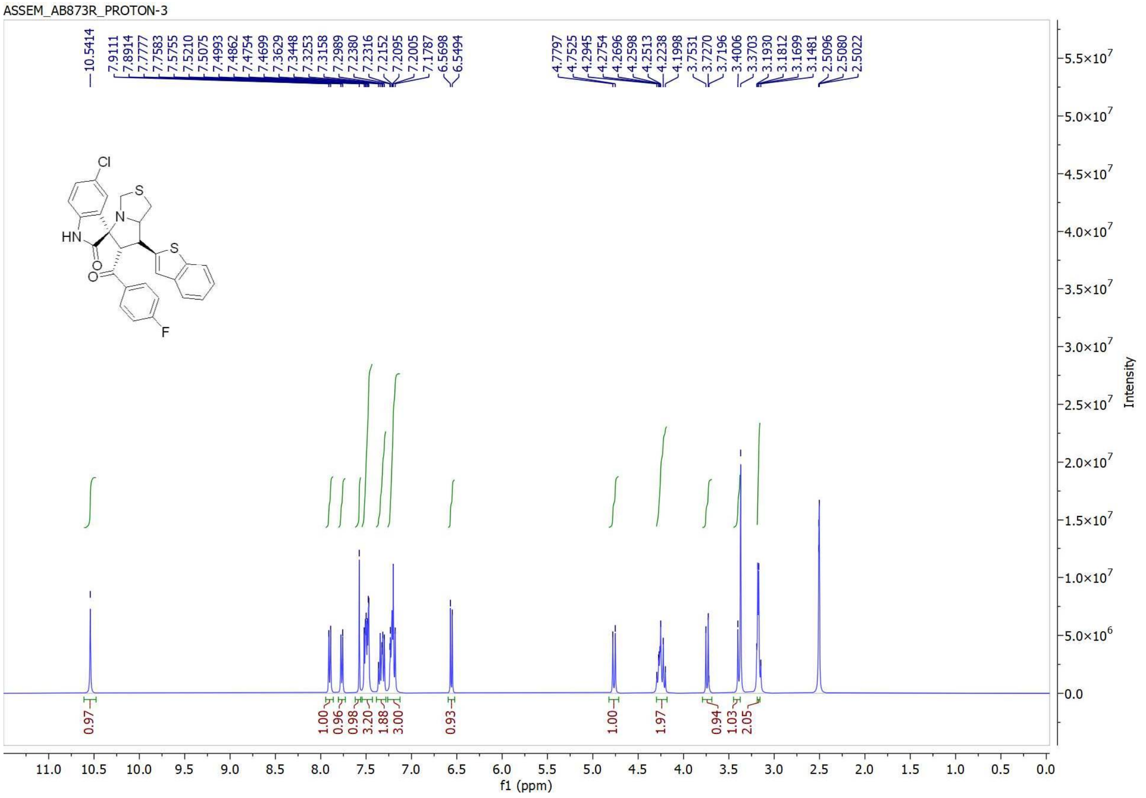

Figure S25: ^{1}HNMR of **IIg**

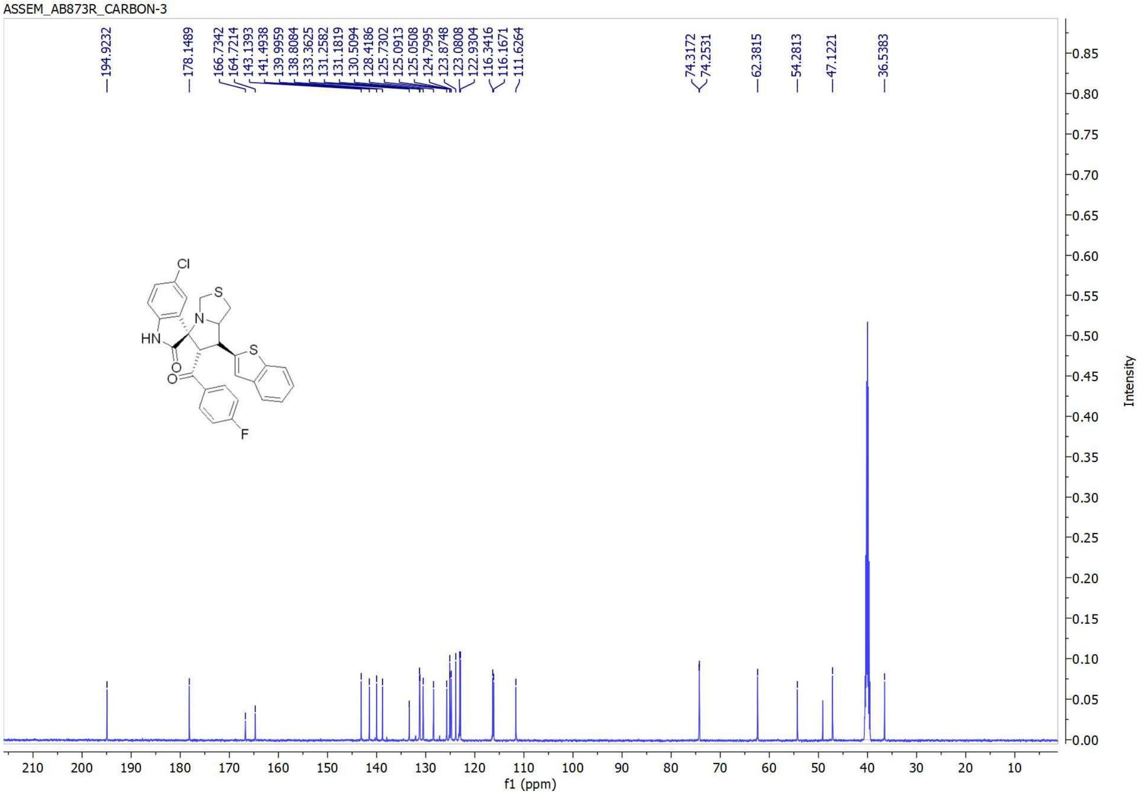

Figure S26: ^{13}CNMR of **IIg**

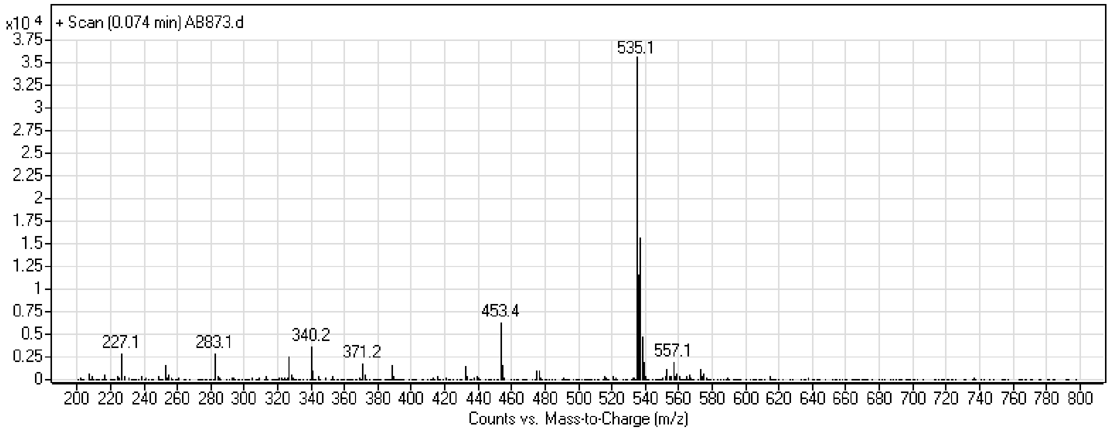

Figure S27: MS of **IIg**

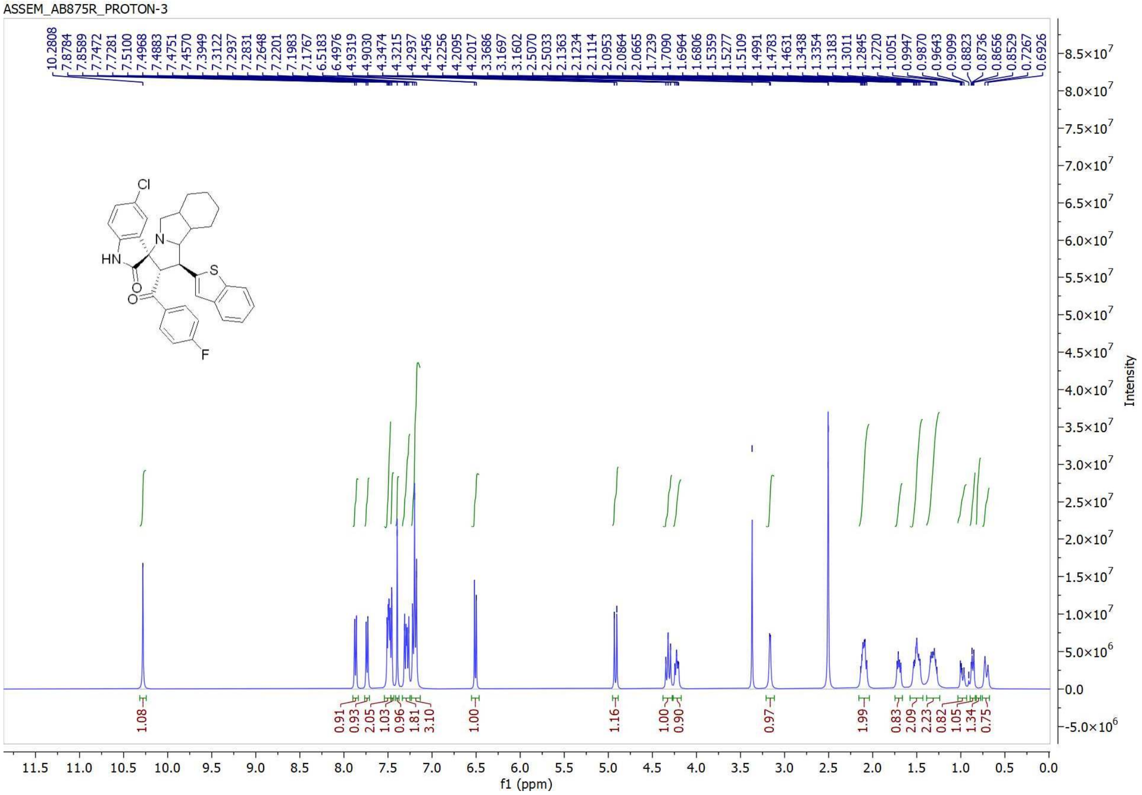

Figure S28: ^{1}HNMR of **IIh**

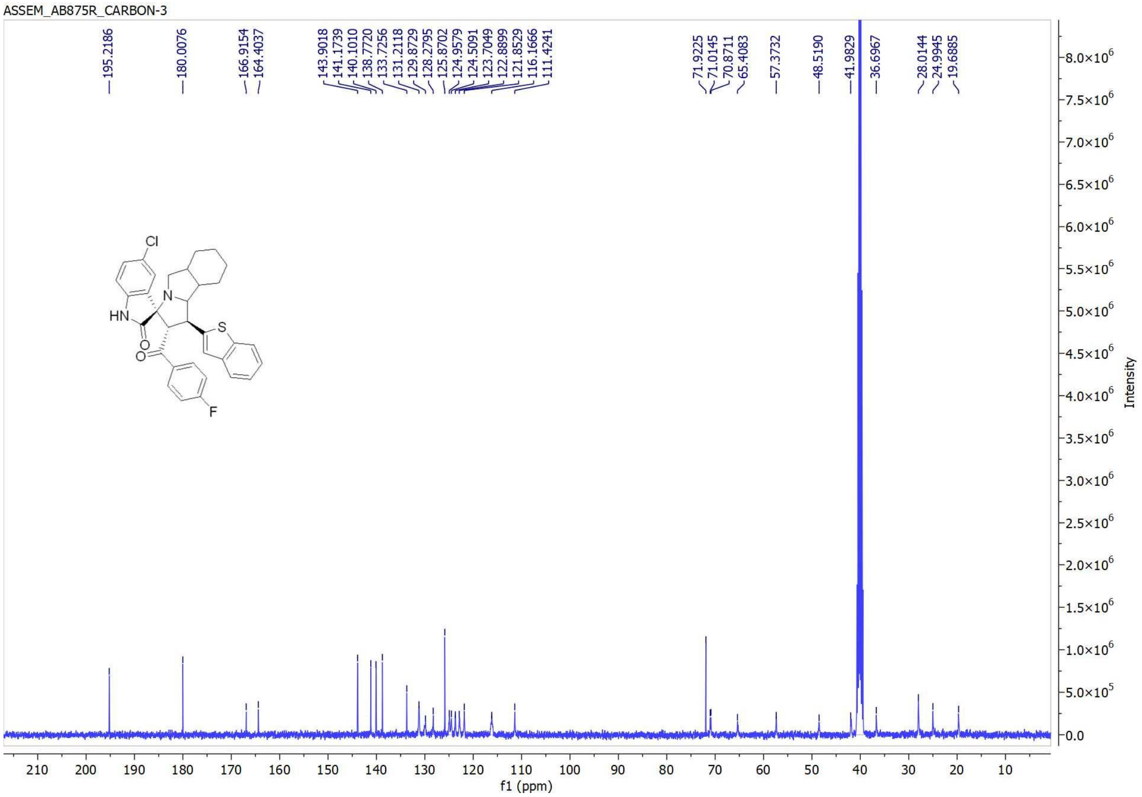

Figure S29: ^{13}CNMR of **IIh**

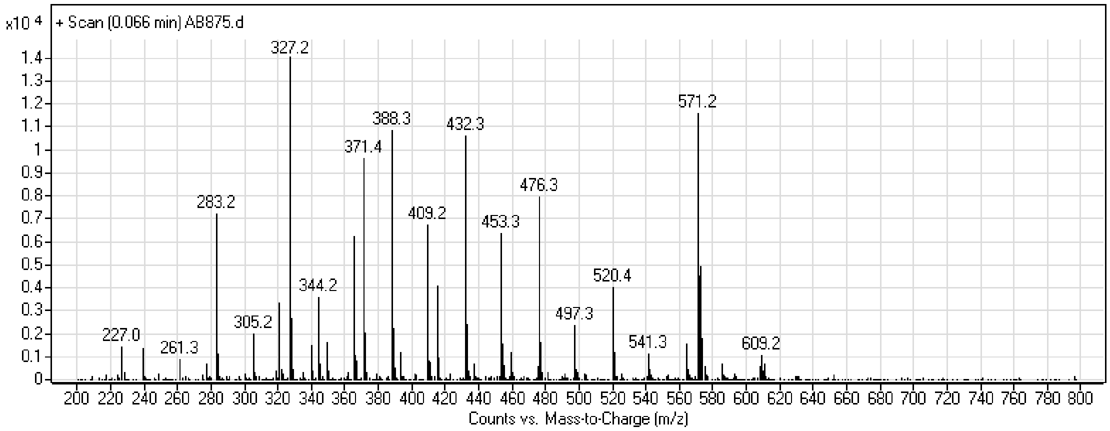

Figure S30: MS of **IIh**

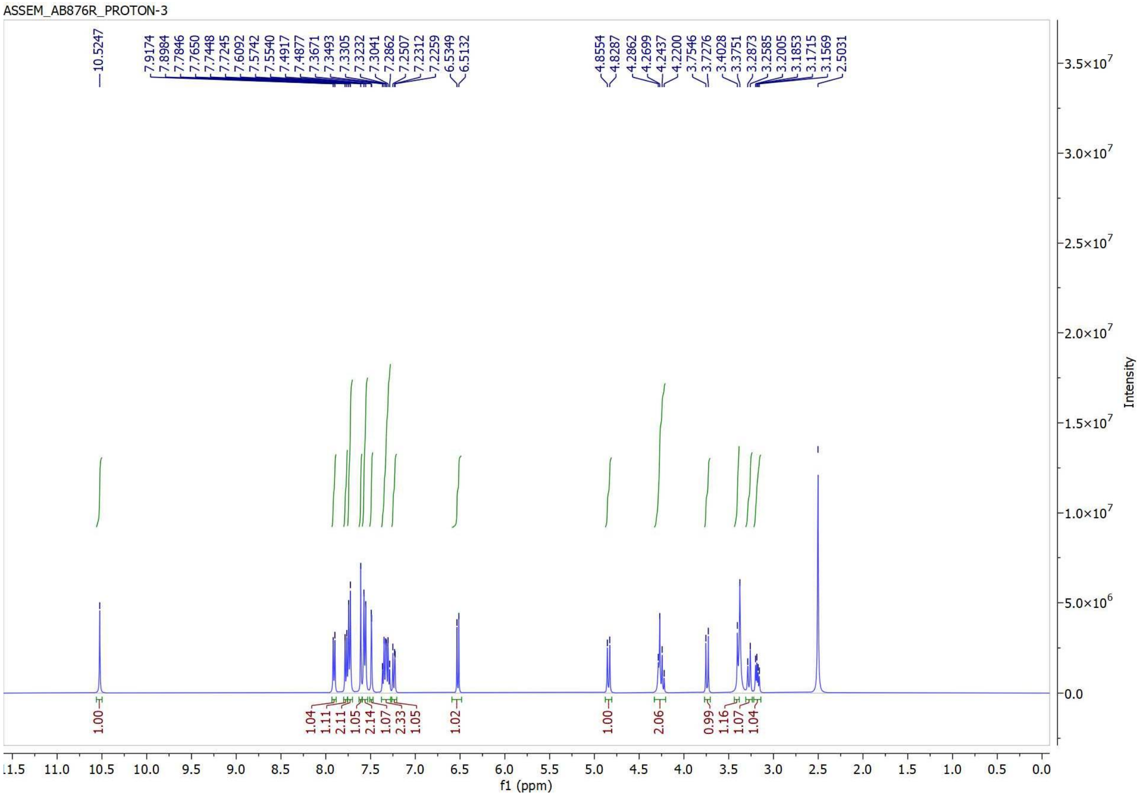

Figure S31: ^{1}HNMR of **IIi**

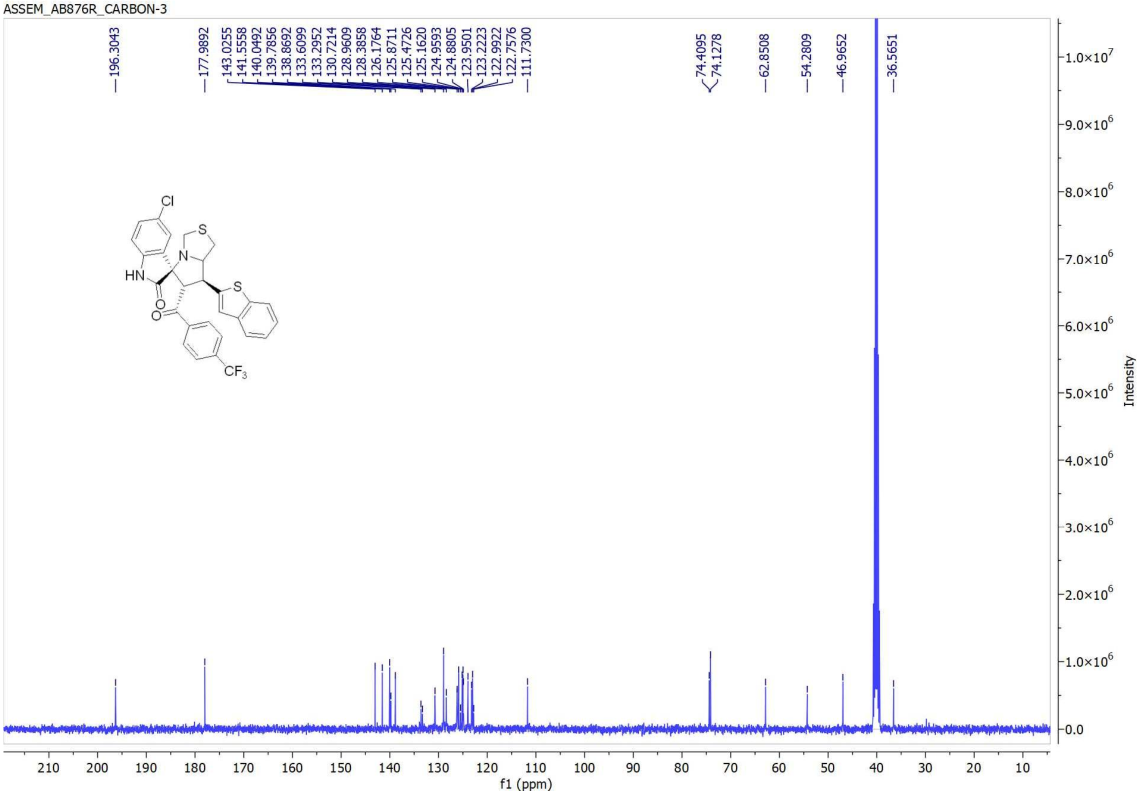

Figure S32: ^{13}CNMR of **IIi**

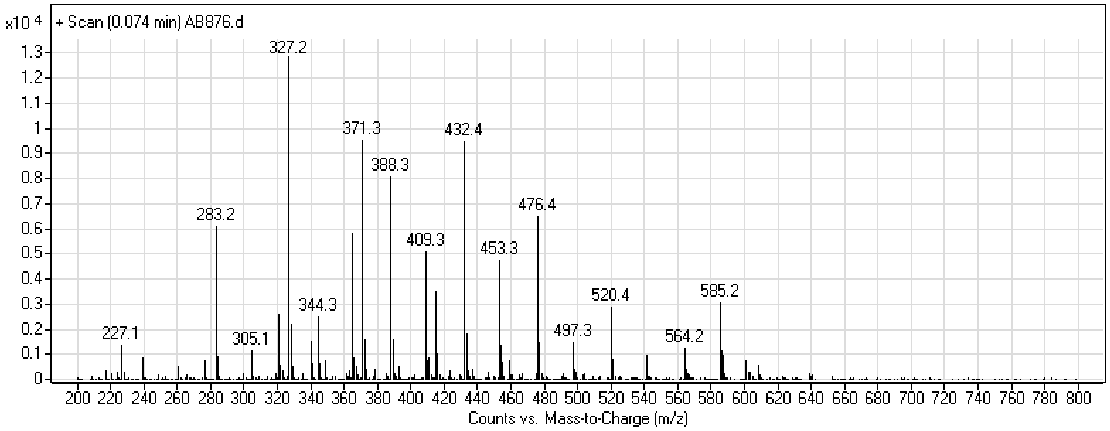

Figure S33: ^{13}CNMR of **IIi**

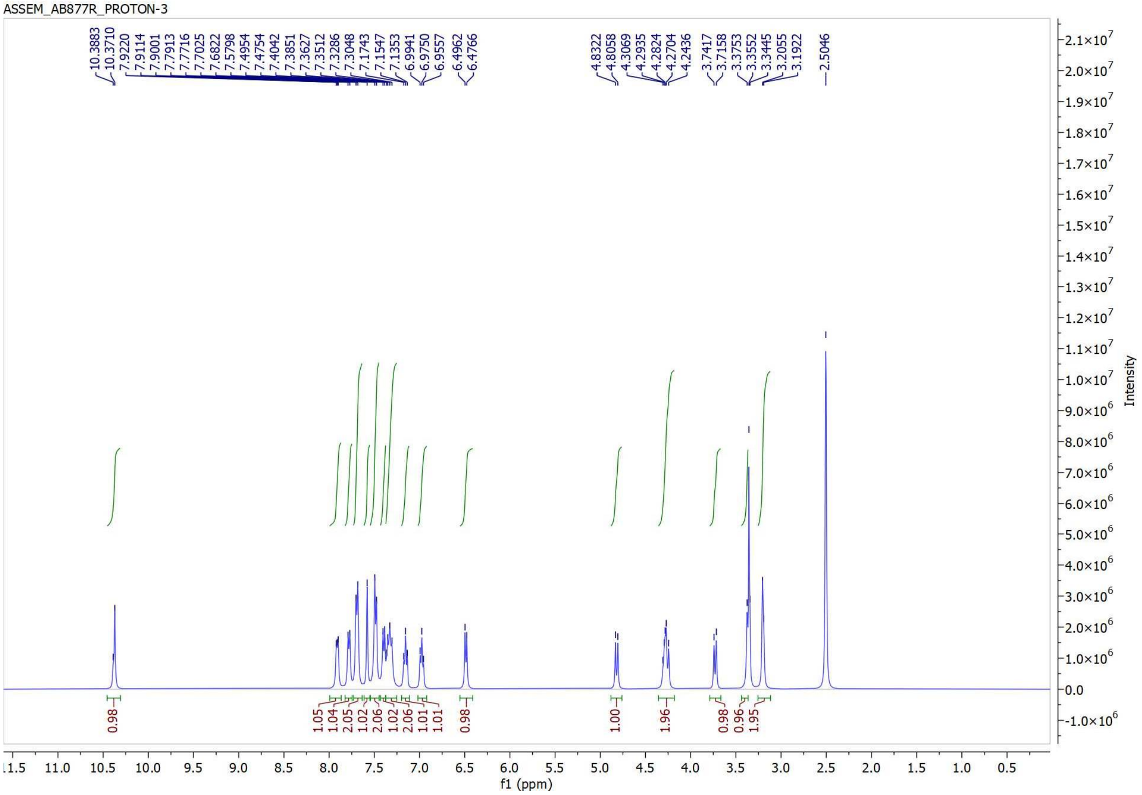

Figure S34: ^{1}HNMR of **IIj**

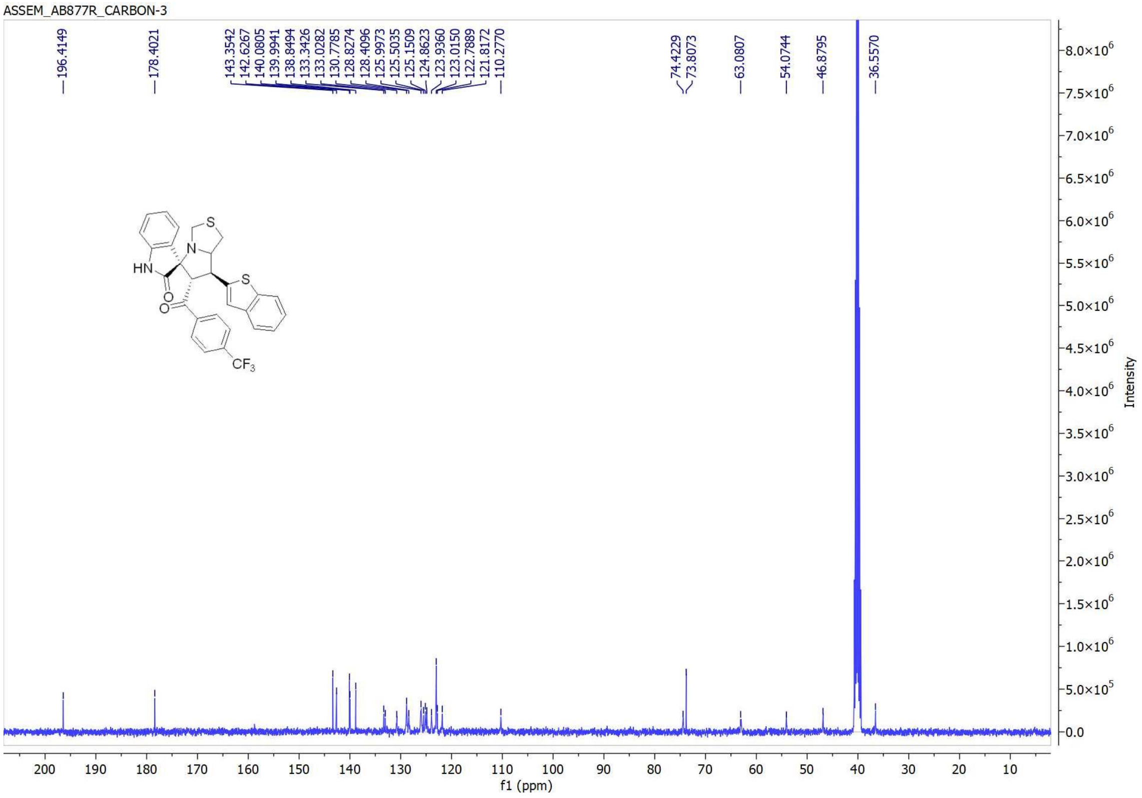

Figure S35: ^{13}CNMR of **IIj**

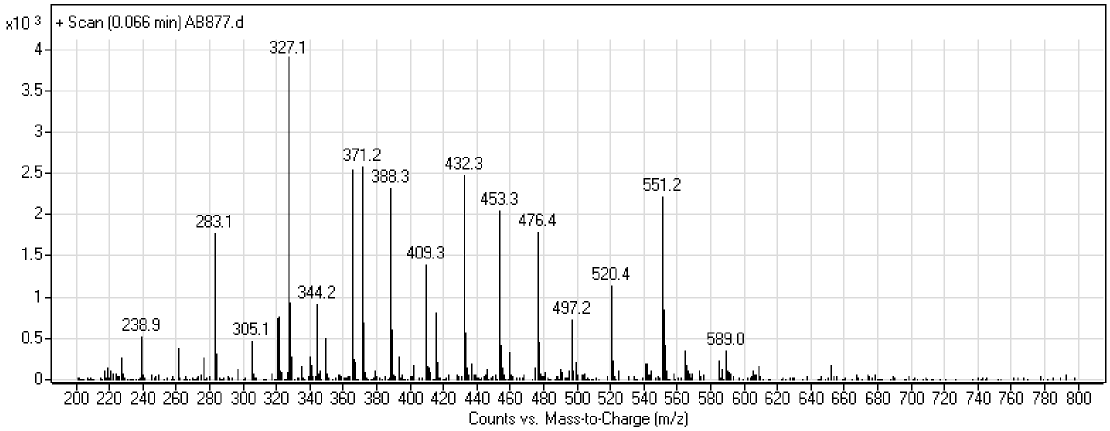

Figure S36: MS of **IIj**

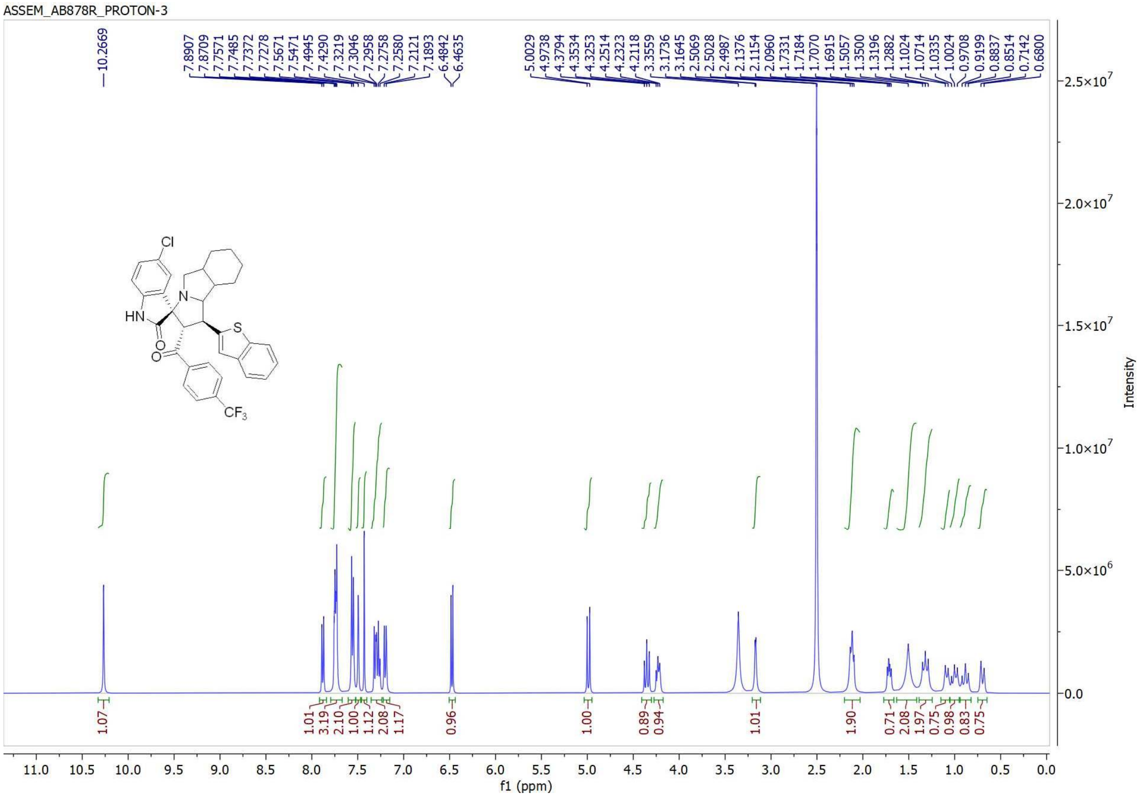

Figure S37: ^{1}HNMR of **IIk**

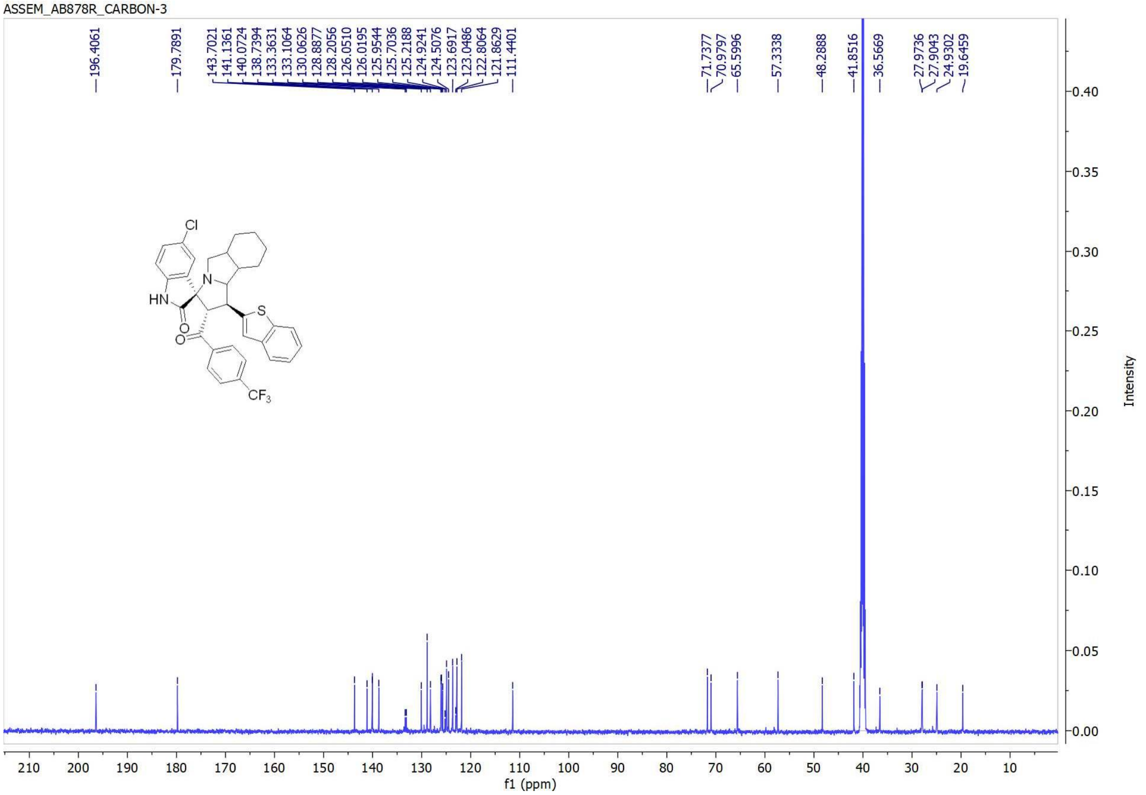

Figure S38: ^{13}CNMR of **IIk**

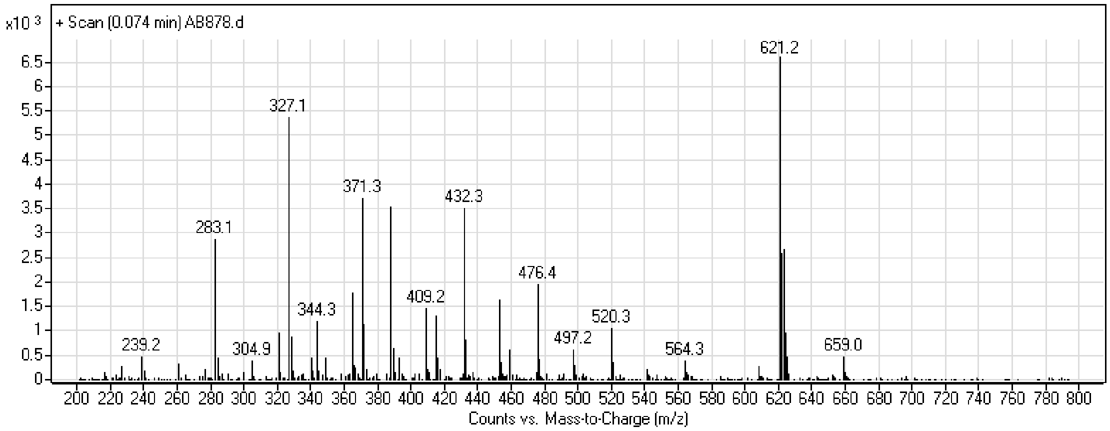

Figure S39: MS of **IIk**

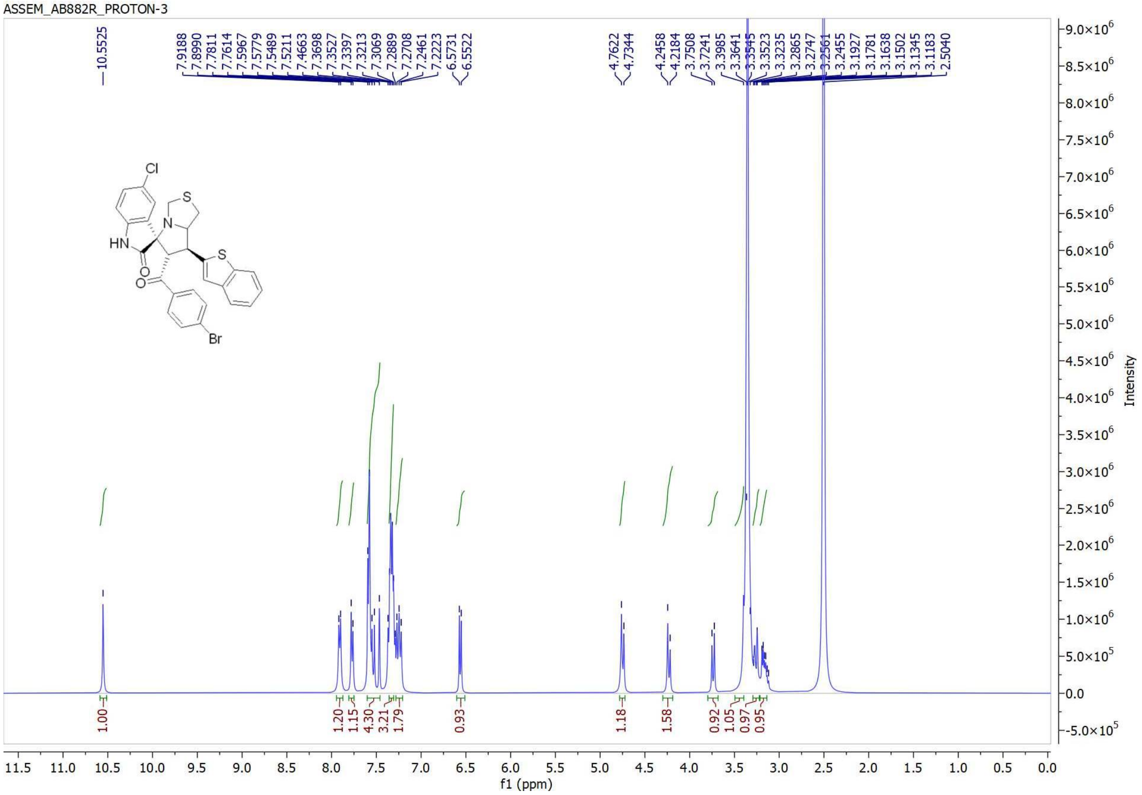

Figure S40: ^{1}HNMR of **IIl**

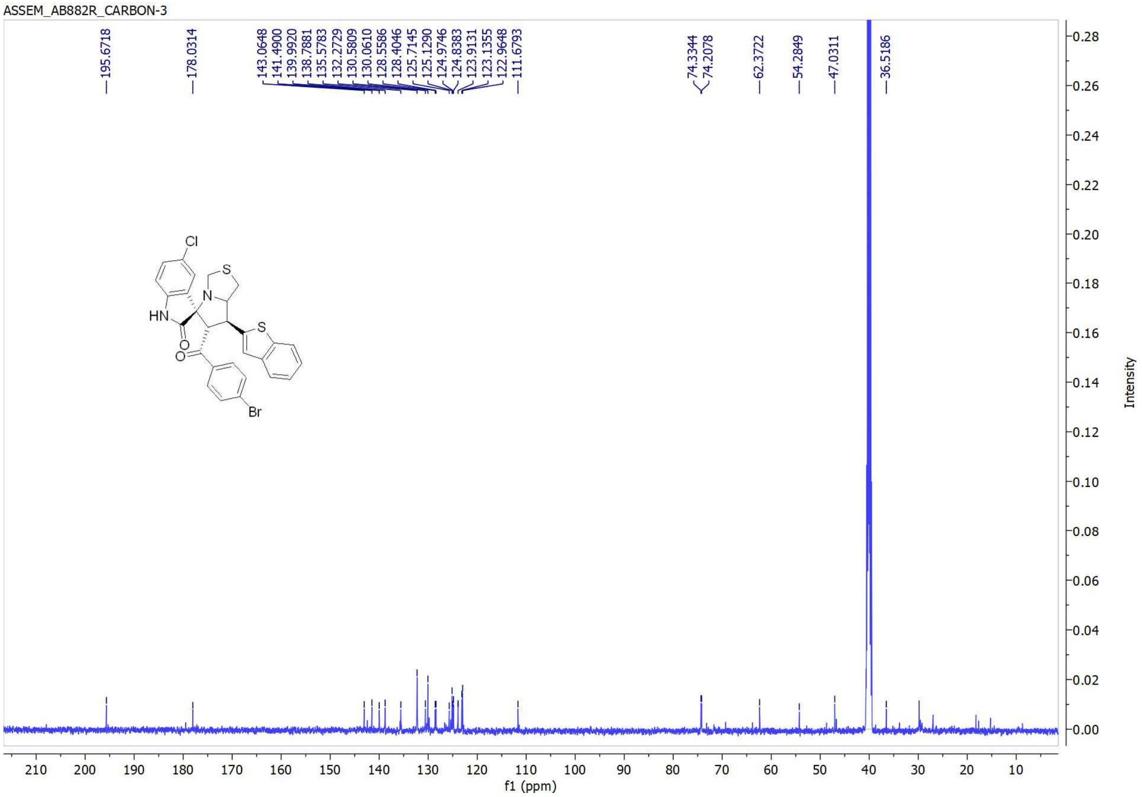

Figure S41: ^{13}CNMR of **IIl**

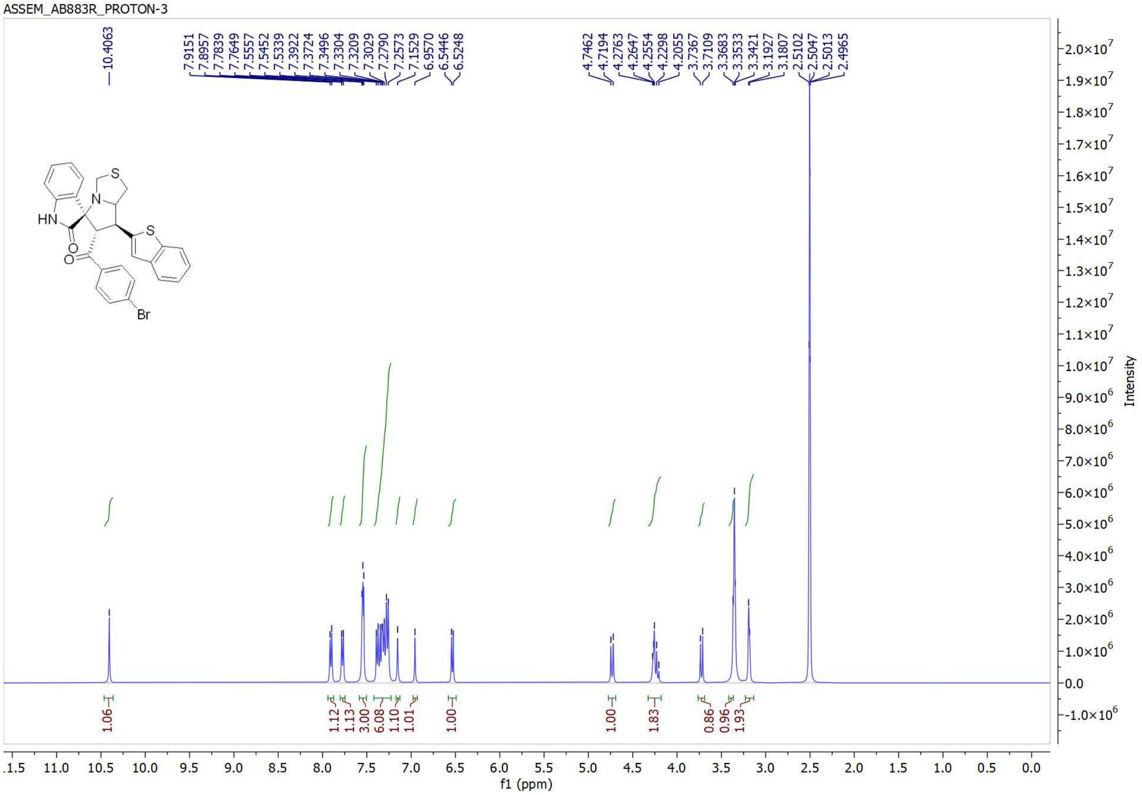

Figure S42: ^{1}HNMR of **IIm**

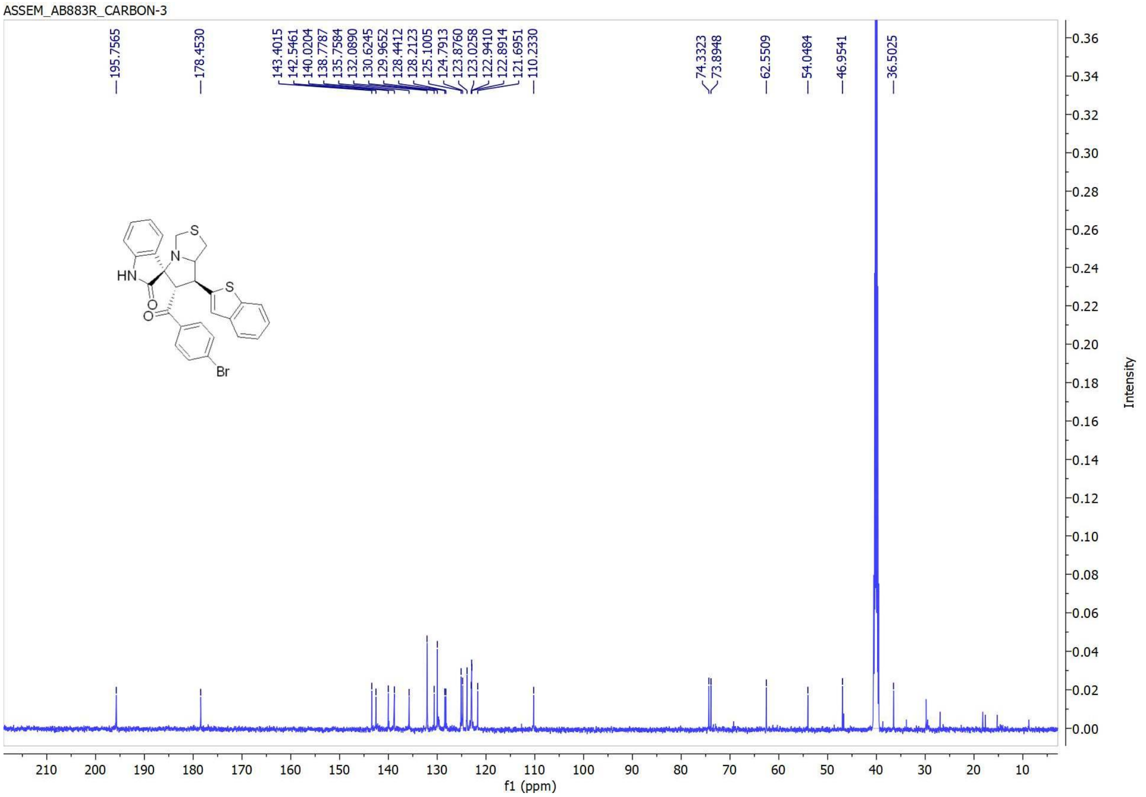

Figure S43: ^{13}CNMR of **IIm**

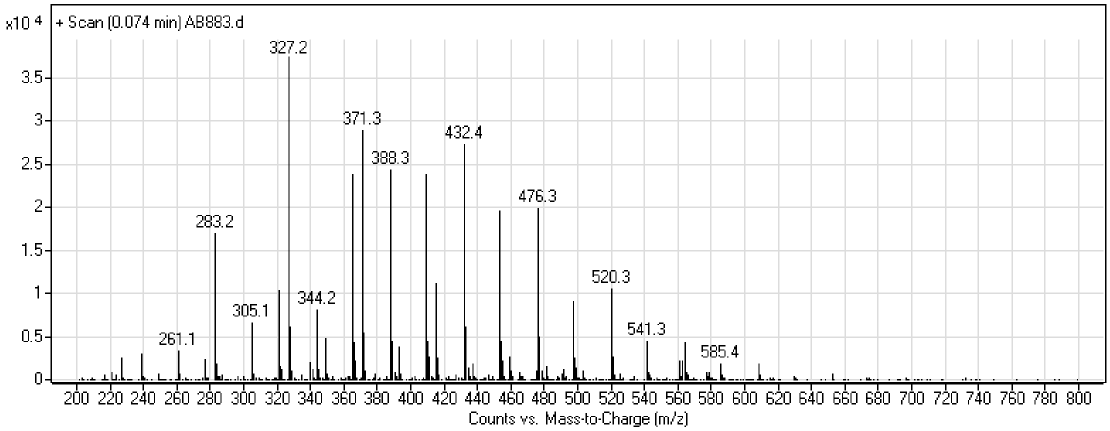

Figure S44: MS of **IIm**

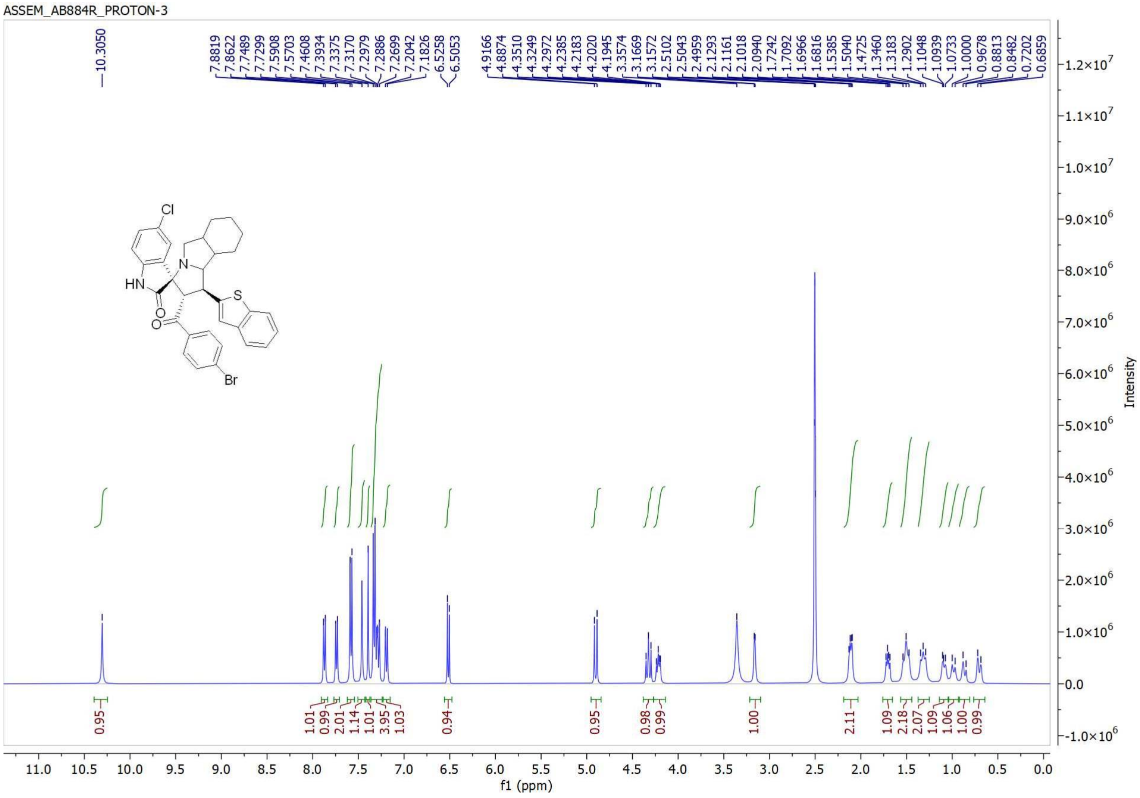

Figure S45: ^{1}HNMR of **IIn**

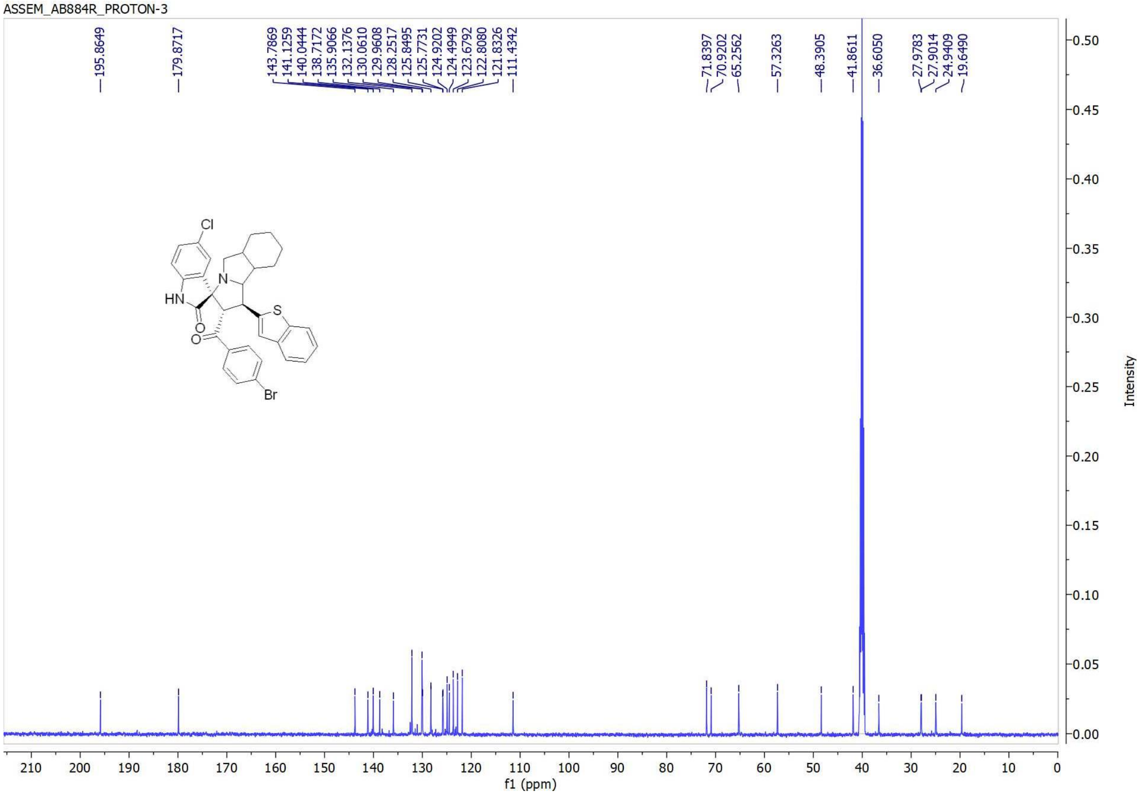

Figure S46: ^{13}CNMR of **IIn**

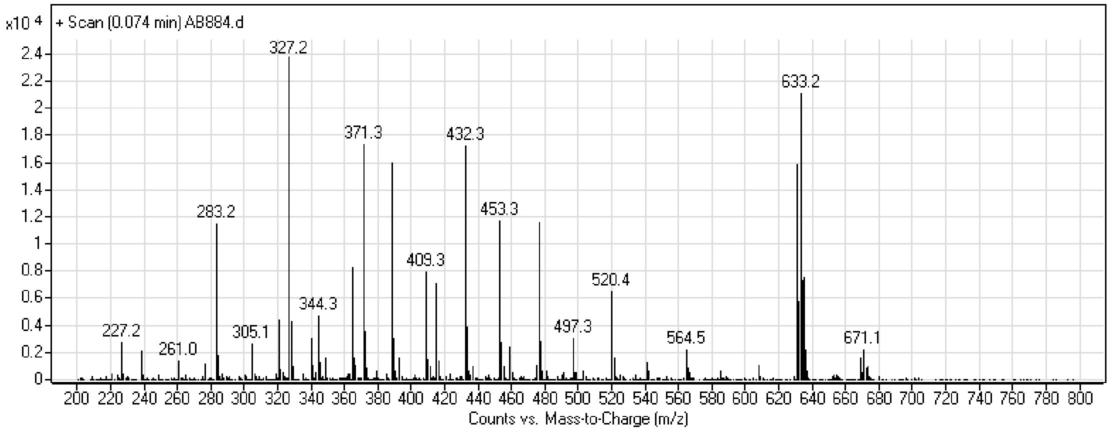

Figure S47: MS of **IIn**

Effect of IIa compound on AchE inhibition

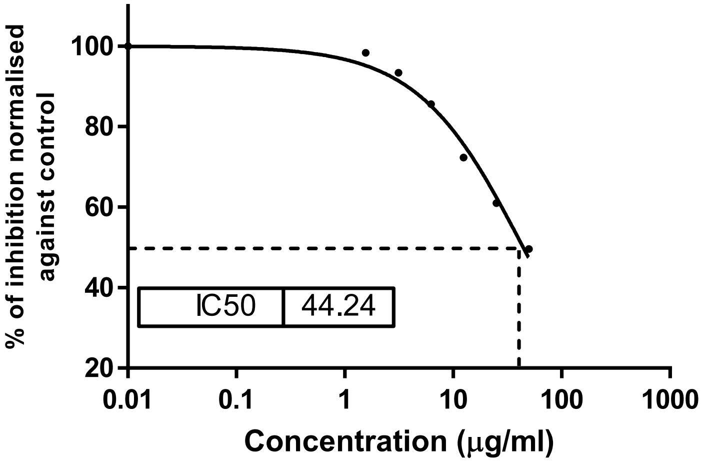

Control Avg OD	0.653					
OD	0.324	0.398	0.472	0.559	0.610	0.642
Conc (ug/ml)	50	25	12.5	6.25	3.125	1.56
Inhibition %	50.38	39.05	27.72	14.40	6.58	1.68
IC50 (ug/ml)	44.24					

Effect of IIb compound on AchE inhibition

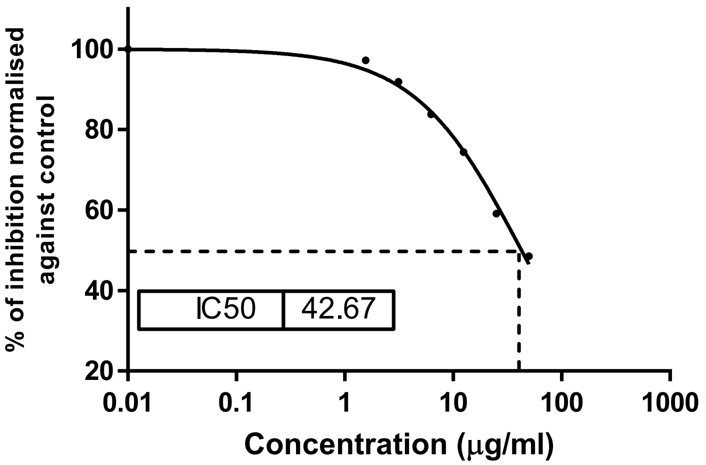

Control Avg OD	0.653					
OD	0.317	0.386	0.486	0.547	0.600	0.635
Conc (ug/ml)	50	25	12.5	6.25	3.125	1.56
Inhibition %	51.45	40.89	25.57	16.23	8.12	2.76
IC50 (ug/ml)	42.67					

Effect of compound IIc on AchE inhibition

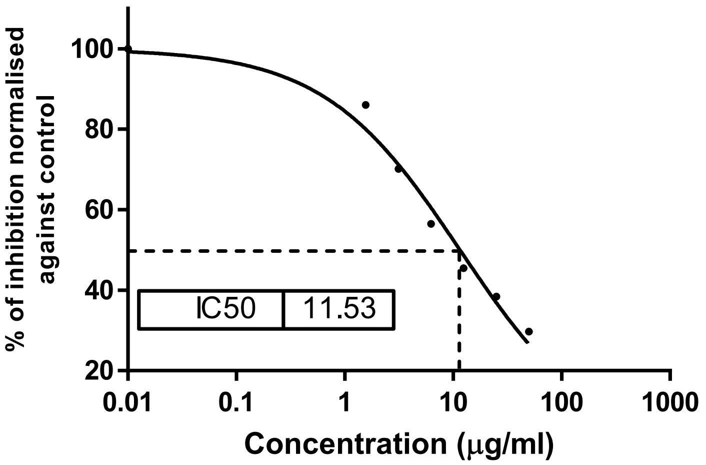

Control Avg OD	0.653					
OD	0.194	0.251	0.297	0.369	0.458	0.562
Conc (ug/ml)	50	25	12.5	6.25	3.125	1.56
Inhibition %	70.29	61.56	54.52	43.49	29.86	13.94
IC50 (ug/ml)	11.53					

Effect of IId compound on AchE inhibition

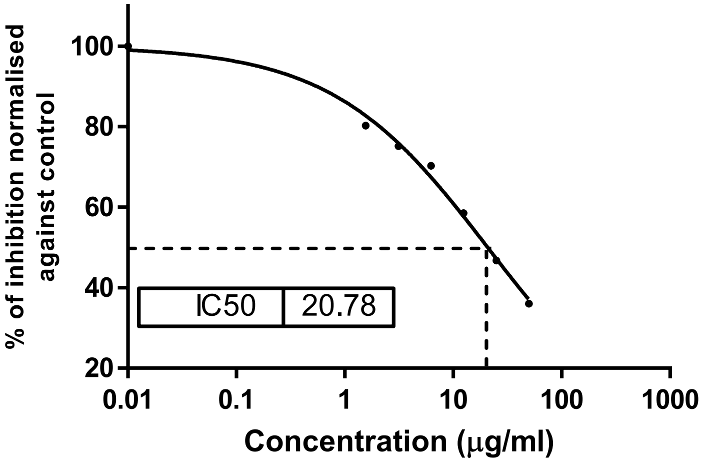

Control Avg OD	0.653				
OD	0.235	0.305	0.382	0.459	0.491	0.524
Conc (ug/ml)	50	25	12.5	6.25	3.125	1.56
Inhibition %	64.01	53.29	41.50	29.71	24.81	19.75
IC50 (ug/ml)	20.78					

Effect of IIe compound on AchE inhibition

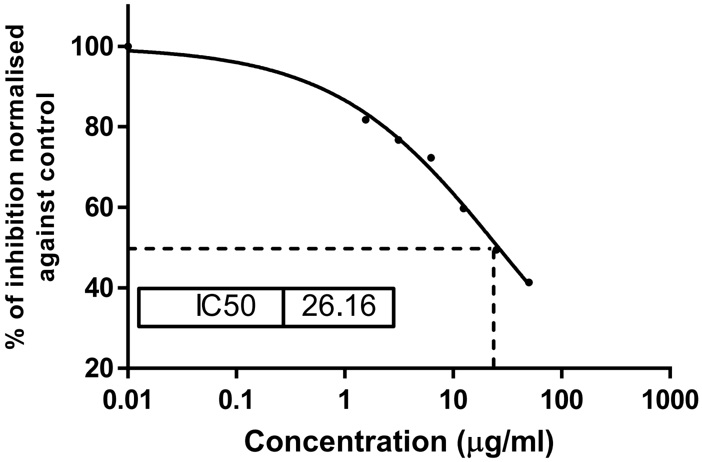

Control Avg OD	0.653					
OD	0.270	0.323	0.390	0.472	0.501	0.534
Conc (ug/ml)	50	25	12.5	6.25	3.125	1.56
Inhibition %	58.65	50.54	40.28	27.72	23.28	18.22
IC50 (ug/ml)	26.16					

Effect of IIf compound on AchE inhibition

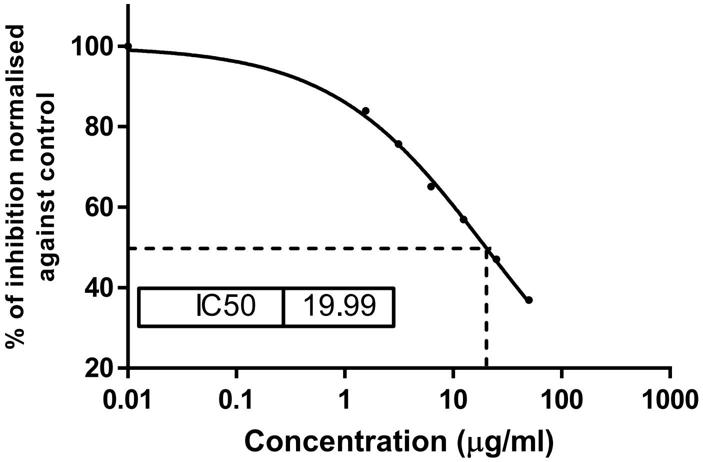

Control Avg OD	0.653					
OD	0.241	0.307	0.372	0.425	0.494	0.548
Conc (ug/ml)	50	25	12.5	6.25	3.125	1.56
Inhibition %	63.09	52.99	43.03	34.92	24.35	16.08
IC50 (ug/ml)	19.99					

Effect of IIg compound on AchE inhibition

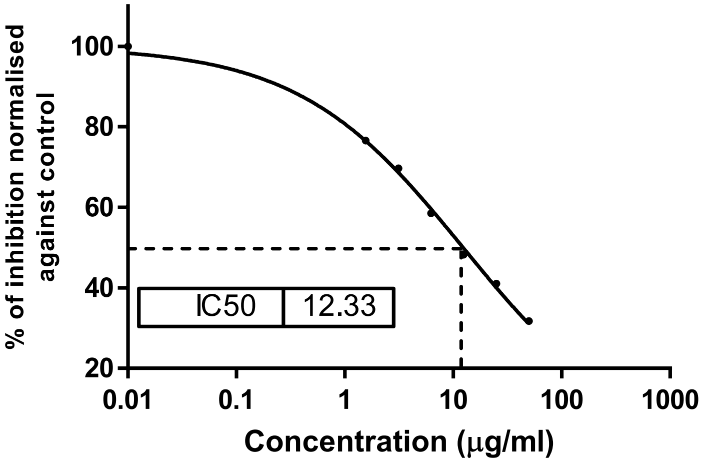

Control Avg OD	0.653					
OD	0.207	0.268	0.315	0.382	0.455	0.500
Conc (ug/ml)	50	25	12.5	6.25	3.125	1.56
Inhibition %	68.30	58.96	51.76	41.50	30.32	23.43
IC50 (ug/ml)	12.33					

Effect of IIh compound on AchE inhibition

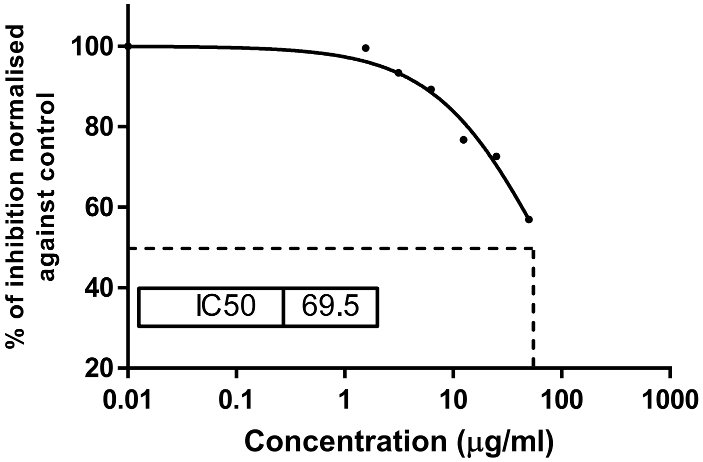

Control Avg OD	0.653					
OD	0.372	0.474	0.501	0.583	0.610	0.650
Conc (ug/ml)	50	25	12.5	6.25	3.125	1.56
Inhibition %	43.03	27.41	23.28	10.72	6.58	0.46
IC50 (ug/ml)	69.5					

Effect of IIi compound on AchE inhibition

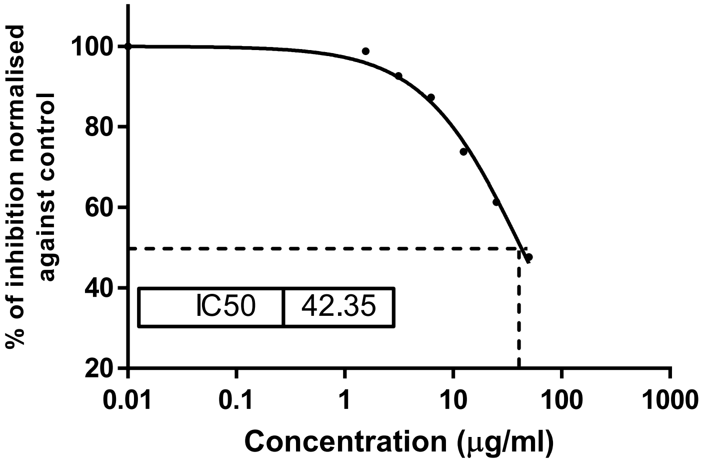

Control Avg OD	0.653					
OD	0.311	0.400	0.482	0.570	0.605	0.645
Conc (ug/ml)	50	25	12.5	6.25	3.125	1.56
Inhibition %	52.37	38.74	26.19	12.71	7.35	1.23
IC50 (ug/ml)	42.35					

Effect of IIj compound on AchE inhibition

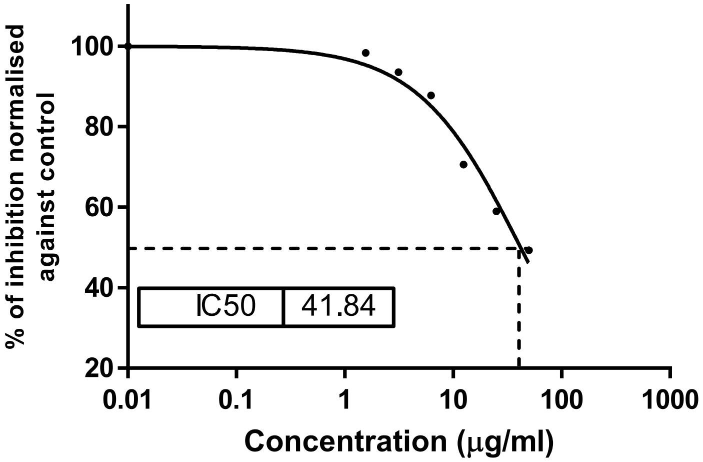

Control Avg OD	0.653					
OD	0.322	0.385	0.461	0.573	0.611	0.642
Conc (ug/ml)	50	25	12.5	6.25	3.125	1.56
Inhibition %	50.69	41.04	29.40	12.25	6.43	1.68
IC50 (ug/ml)	41.84					

Effect of IIk compound on AchE inhibition

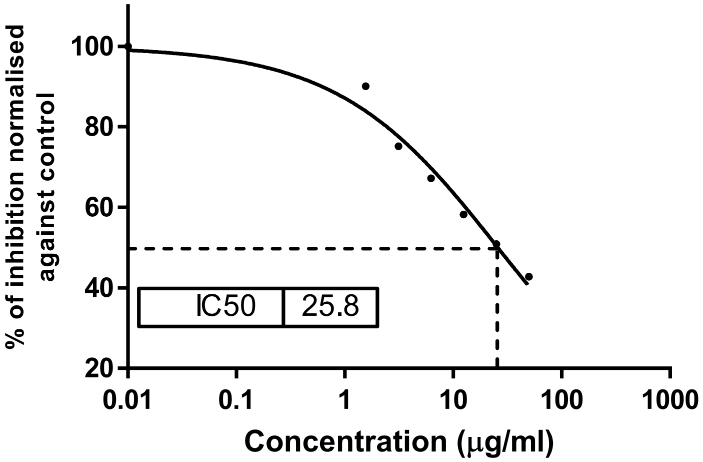

Control Avg OD	0.653					
OD	0.279	0.332	0.380	0.439	0.491	0.588
Conc (ug/ml)	50	25	12.5	6.25	3.125	1.56
Inhibition %	57.27	49.16	41.81	32.77	24.81	9.95
IC50 (ug/ml)	25.8					

Effect of IIl compound on AchE inhibition

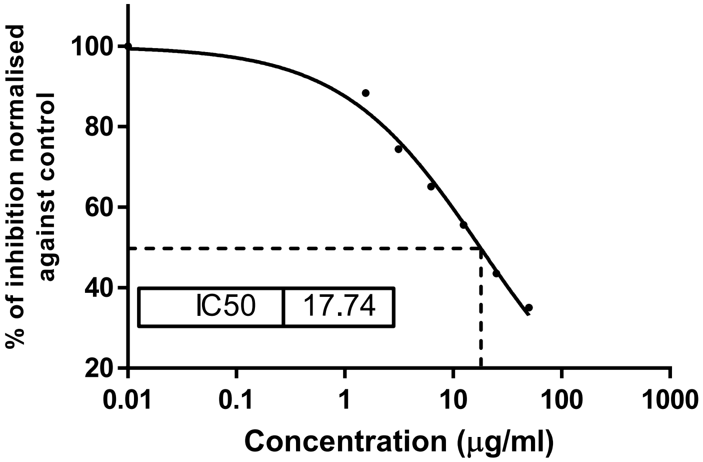

Control Avg OD	0.653					
OD	0.229	0.284	0.363	0.425	0.486	0.577
Conc (ug/ml)	50	25	12.5	6.25	3.125	1.56
Inhibition %	64.93	56.51	44.41	34.92	25.57	11.64
IC50 (ug/ml)	17.74					

Effect of IIm compound on AchE inhibition

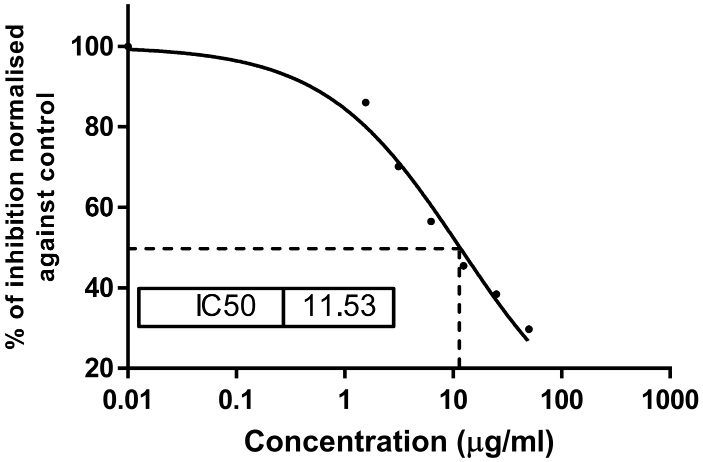

Control Avg OD	0.653					
OD	0.194	0.251	0.297	0.369	0.458	0.562
Conc (ug/ml)	50	25	12.5	6.25	3.125	1.56
Inhibition %	70.29	61.56	54.52	43.49	29.86	13.94
IC50 (ug/ml)	11.53					

Effect of IIn compound on AchE inhibition

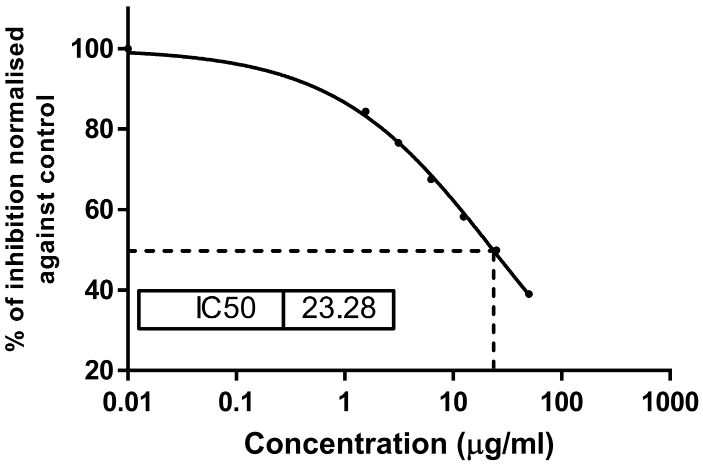

Control Avg OD	0.653					
OD	0.255	0.326	0.380	0.441	0.500	0.551
Conc (ug/ml)	50	25	12.5	6.25	3.125	1.56
Inhibition %	60.95	50.08	41.81	32.47	23.43	15.62
IC50 (ug/ml)	23.28					
